# Supplementary figures and images for: ALIX and ESCRT-III Coordinately Control Cytokinetic Abscission during Germline Stem Cell Division In Vivo
Source: PLoS Genet. 2015 Jan 30;11(1):e1004904. doi: 10.1371/journal.pgen.1004904 (PMC4312039; doi:10.1371/journal.pgen.1004904)

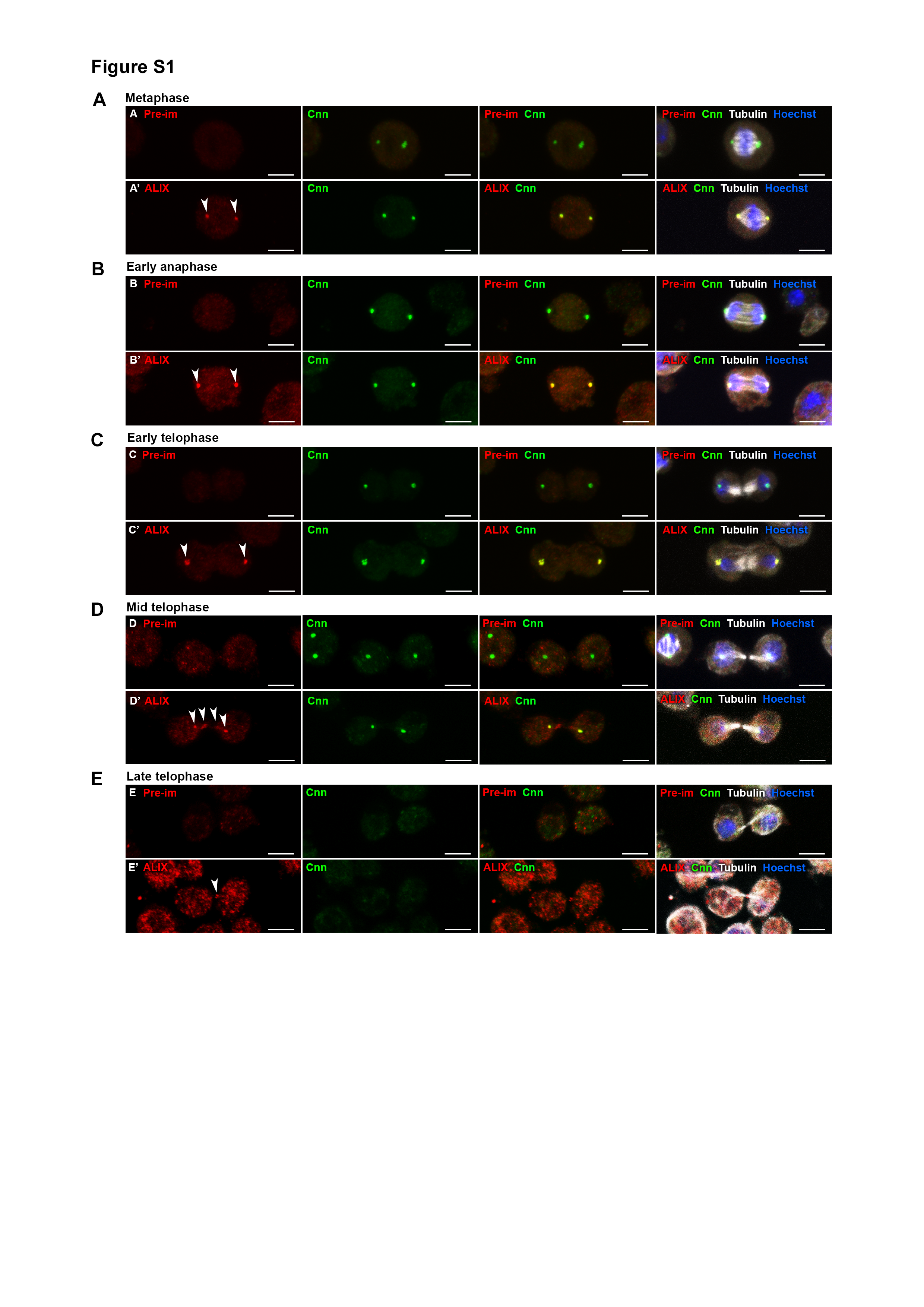

Supplement: S1 Fig — (A-C) ALIX co-localizes with Centrosomin (Cnn) at centrosomes in meta-, anaphase and early telophase (A’, B’ and C’). No signal is detected at centrosomes using the pre-immune (pre-im) serum (A, B and C). (D) ALIX co-localizes with Cnn at centrosomes and in addition appears at the intercellular bridge in mid telophase where it overlaps with the mitotic spindle (D’). No signal is detected at centrosomes nor at the intercellular bridge using the pre-immune serum (D). (E) In late telophase/cytokinesis, ALIX localizes to the dark region in the α-tubulin staining at the centre of the intercellular bridge, indicating its localization at the midbody ring (E’). ALIX also shows a vesicular pattern within the cell at this stage (E’). No signal is detected at the midbody ring using the pre-immune serum (E). A weak vesicular pattern detected, but is much weaker than in (E’). In all panels, S2 cells were fixed and stained with antibodies against ALIX (red), Cnn (green) and α-tubulin (white), and with Hoechst (blue). Images in all panels were captured with the same intensity. Scale bars represent 5 µm. (TIF) [file pgen.1004904.s001.tif]

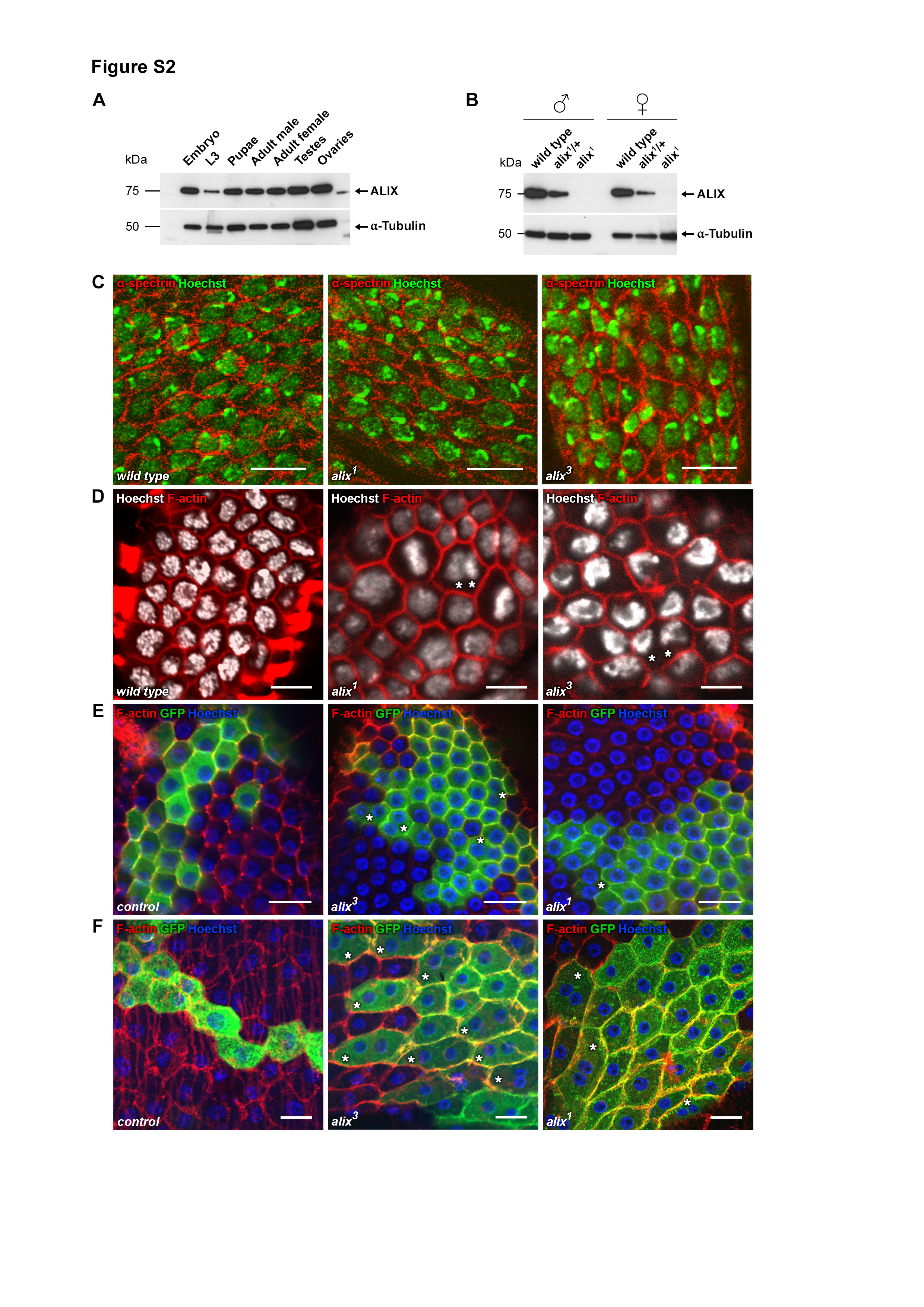

Supplement: S2 Fig — (A) Western blot showing ALIX expression levels in Drosophila embryos, L3 larvae, pupae, adult males and females as well as testes and ovaries. α-tubulin was used as a loading control. (B) Western blot showing loss of ALIX protein in alix1 mutant males and females. Heterozygote alix1/TM6B, Tb males and females show reduced protein levels compared to wild type. α-tubulin was used as a loading control. (C) Left: Image of wild type stage 16 embryonic epithelium. Middle and right: Images of homozygous alix1 and alix3 mutant stage 16 embryonic epithelia. Embryos were fixed and stained with antibodies against α-spectrin (red) and with Hoechst (green). More than 1000 cells from five embryos of each genotype were analyzed for the presence of mono- and bi-nucelate cells and no evident bi-nucleation could be detected for any of the genotypes. Scale bars represent 10 µm. (D) Left: Image of wild type follicle cell epithelium of stage 6 egg chamber. Middle and right: Images of alix1 and alix3 mutant follicle cell epithelia of stage 6 egg chambers. Bi-nucleate cells are indicated with asterisks. Ovaries were fixed and stained to visualize F-actin (red) and nuclei (white, Hoechst). Scale bars represent 5 µm. See also S1 Table. (E) Left: Stage 10 EC with GFP-positive mono-nucleate control follicle cell clones. Middle and right: alix3 and alix1 mutant GFP-positive clones with bi-nucleate cells (asterisks). Ovaries were fixed and stained to visualize F-actin (red) and nuclei (Hoechst, blue). Scale bars represent 20 µm. See also S2 Table. (F) Left: Stage 14 EC with GFP-positive mononucleate control follicle cell clones. Middle and right: alix3 and alix1 mutant GFP-positive clones with bi-nucleate cells (asterisks). The bi-nucleation in the alix mutant clones may arise via alternative mechanisms. It is possible that loss of ALIX function leads to loss of the connection of the stable intercellular bridge between follicle cells with the plasma membrane as the egg chamber develops from st [file pgen.1004904.s002.tif]

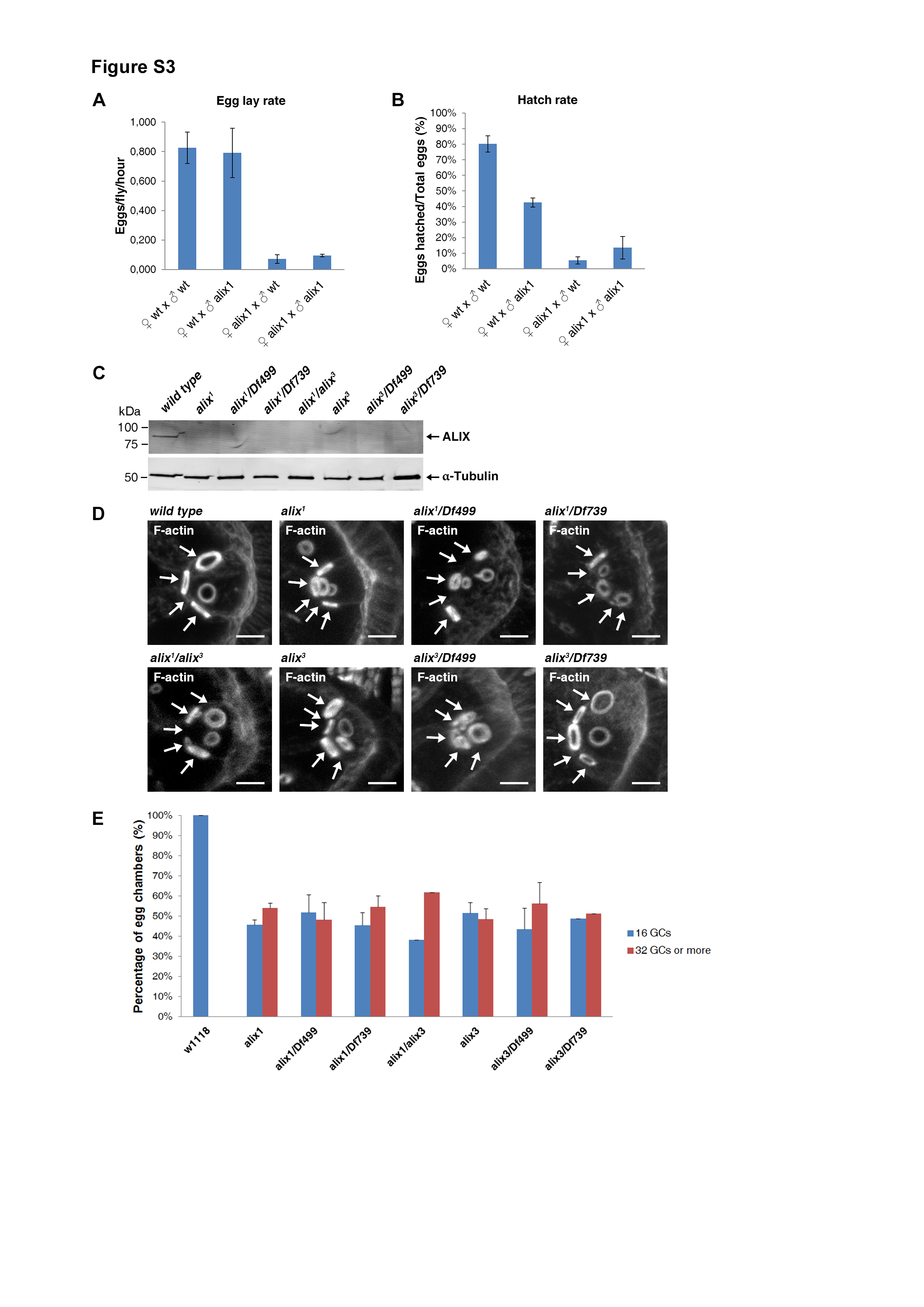

Supplement: S3 Fig — (A) Graph showing the average egg lay rates for wild type and alix1 mutant females crossed to either wild type or alix1 mutant males from three independent experiments. Data are presented as mean ± STD. (B) Graph showing average hatch rates for the eggs laid in the crosses from the three independent experiments in (B). Data are presented as mean ± STD. (C) Western blot showing expression of ALIX protein in wild type ovaries and loss of ALIX protein in ovaries of alix1 homozygote mutant females, of females in which the alix1 allele is combined with two different deficiences (alix1/Df(3R)BSC499, alix1/Df499 and alix1/Df(3R)BSC739, alix1/Df739) or with the alix3 allele (alix1/alix3), of alix3 homozygote mutant females, or females in which the alix3 allele is combined with the two different deficiences (alix3/Df499 and alix3/Df739). α-tubulin served as a control for protein loading. (D) Images showing four ring canals (arrows) to the oocyte in a wild type egg chamber and five ring canals (arrows) to the oocyte in egg chambers of the genotypes in (C). Ovaries were fixed and stained to visualize F-actin (white). Scale bars represent 5 µm. (E) Graph showing the percentages of egg chambers with 16, 32 or more germ cells the indicated genotypes in (C-D). Wild type, three independent experiments, n = 362 egg chambers; alix1, two independent experiments, n = 154 egg chambers; alix1/Df(3R)BSC499, two independent experiments, n = 242 egg chambers; alix1/Df(3R)BSC739, two independent experiments, n = 38; alix1/alix3, one experiment, n = 42 egg chambers, alix3, two independent experiments, n = 150 egg chambers, alix3/Df(3R)BSC499, two independent experiments, n = 139; alix3/Df(3R)BSC739, one experiment, n = 88 egg chambers. Data are presented as mean ± STD. (TIF) [file pgen.1004904.s003.tif]

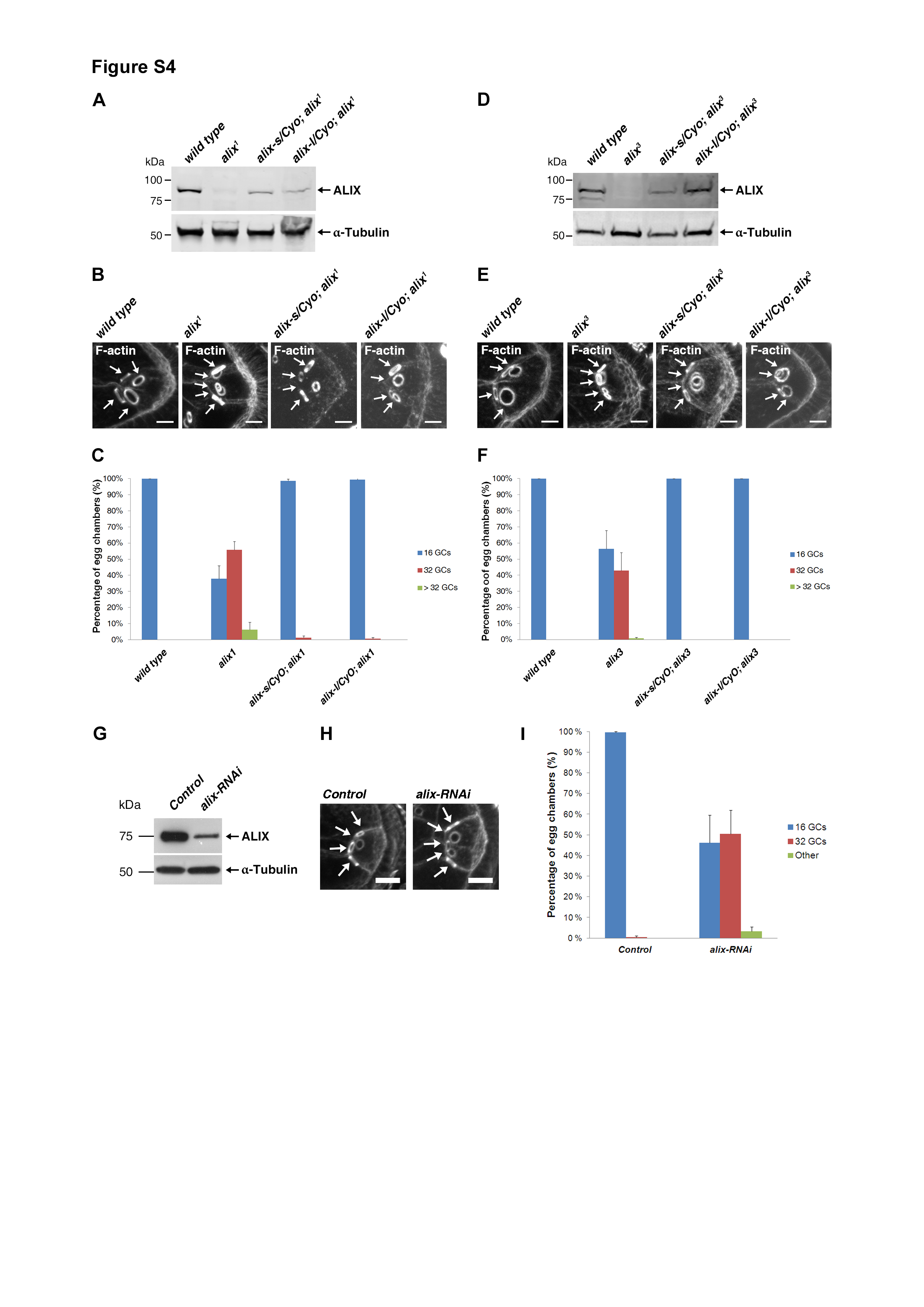

Supplement: S4 Fig — (A) Western blot showing ALIX expression in wild type ovaries, lack of ALIX protein in alix1 homozygote mutant ovaries and ALIX expression in ovaries of two lines with one copy of either of the two genomic rescue constructs (short-alix-rescue, alix-s; long alix-rescue, alix-l) in the alix1 mutant background (alix-s/CyO; alix1 and alix-l/CyO; alix1). Levels of α-tubulin show equal protein loading. Rescue constructs and lines are described in the Materials and Methods. (B) Images showing four ring canals (arrows) to the oocyte in a wild type egg chamber, five ring canals to the oocyte (arrows) in an alix1 mutant egg chamber and four ring canals (arrows) to the oocyte upon reexpression of ALIX in the alix1 mutant background from either of the two rescue constructs (alix-s/CyO; alix1 and alix-l/CyO; alix1). Ovaries were fixed and stained to visualize F-actin (white). Images are planar projections of several sections of a z-stack. Scale bars represent 5 µm. (C) Graph showing the average percentage of egg chambers with 16, 32 or more germ cells of the genotypes in (A) and (B). Wild type, three independent experiments, n = 326 egg chambers; alix1, three independent experiments, n = 236 egg chambers; alix-s/CyO; alix1, three independent experiments, n = 301 egg chambers; alix-l/CyO; alix1, three independent experiments, n = 226 egg chambers. Data are presented as mean ± STD. (D) Western blot showing ALIX expression in wild type ovaries, lack of ALIX protein in alix3 homozygote mutant ovaries and ALIX expression in ovaries from two lines with one copy of either of the two genomic rescue constructs described in (A) in the alix3 mutant background (alix-s/CyO; alix3 and alix-l/CyO; alix3). Levels of α-tubulin show equal protein loading. (E) Images showing four ring canals to the oocyte (arrows) in a wild type egg chamber, five ring canals (arrows) to the oocyte in an alix3 mutant egg chamber and four ring canals (arrows) to the oocyte upon reexpression of ALIX in the alix3 muta [file pgen.1004904.s004.tif]

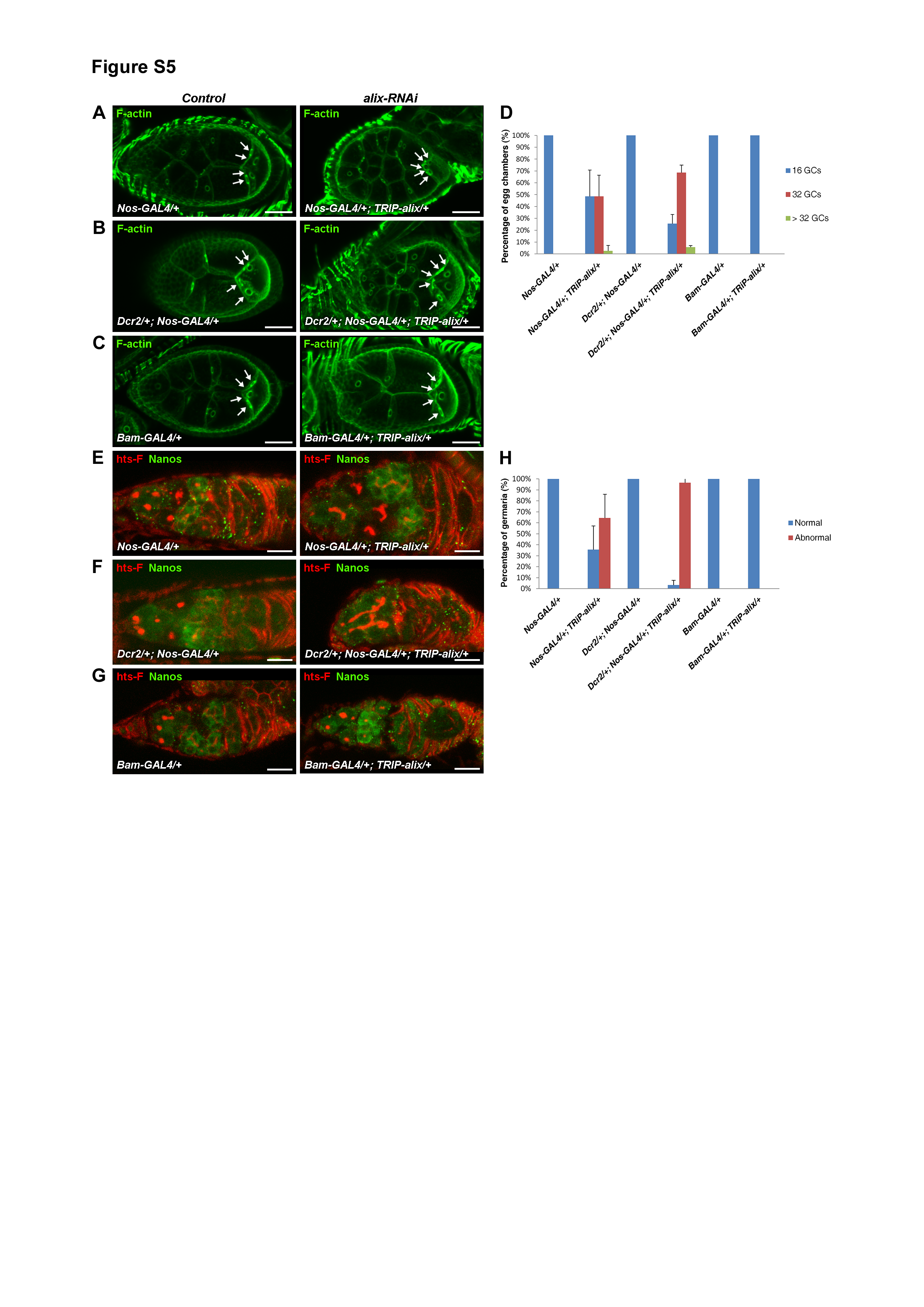

Supplement: S5 Fig — (A) Control (Nos-GAL4/+) egg chamber with four ring canals (arrows) to the oocyte (left image) and egg chamber with alix-RNAi (Nos-GAL4/+; TRiP-alix/+) expression in fGSCs (right image) with five ring canals (arrows) to the oocyte are shown. Nos, Nanos; TRiP-alix = alix-RNAi line from TRiP. (B) Control (Dcr2/+; Nos-GAL4/+) egg chamber with four ring canals (arrows) to the oocyte (left image) and egg chamber with alix-RNAi and Dicer (Dcr2/+; Nos-GAL4/+; TRiP-alix/+) expression (right image) with five ring canals to the oocyte are shown. Dcr2, Dicer 2. (C) Egg chambers from females with the genotypes Bam-GAL4/+ (left image) and Bam-GAL4/TRiP-alix (right image) with four ring canals (arrows) to the oocyte are shown. In (A-C) ovaries were fixed and stained with fluorescently labeled phalloidin (green) and with Hoechst (not shown). Scale bars represent 20 μm. (D) Graph showing the average percentage of egg chambers with 16 or 32 or more germ cells for the genotypes in (A-C). Nos-Gal4/+, three independent experiments, n = 181 egg chambers; Nos-Gal4/+; TRiP-alix/+, three independent experiments, n = 159 egg chambers; Dcr2/+; Nos-GAL4/+, three independent experiments, n = 149 egg chambers; Dcr2/+; Nos-GAL4/+; TRiP-alix /+, three independent experiments, n = 146; Bam-GAL4/+, three independent experiments, n = 179 egg chambers; Bam-GAL4/TRiP-alix, three independent experiments, n = 192 egg chambers. Data are presented as mean ± STD. (E) Germarium from control female (Nos-GAL4/+) with Nanos-positive fGSCs with spectrosomes (left image) and germarium with alix-RNAi expression in germ cells, including fGSCs, using Nos-GAL4 (Nos-GAL4/+; TRiP-alix/+) (right image) are shown. In the right image an fGSC interconnected to three other Nanos-positive cells via fusome is seen in the anterior tip of the germarium. (F) Germarium from control female (Dcr2/+; Nos-GAL4/+) (left image) with Nanos-positive fGSCs with spectrosomes and germarium in which alix-RNAi and Dicer were expressed (Dcr2/ [file pgen.1004904.s005.tif]

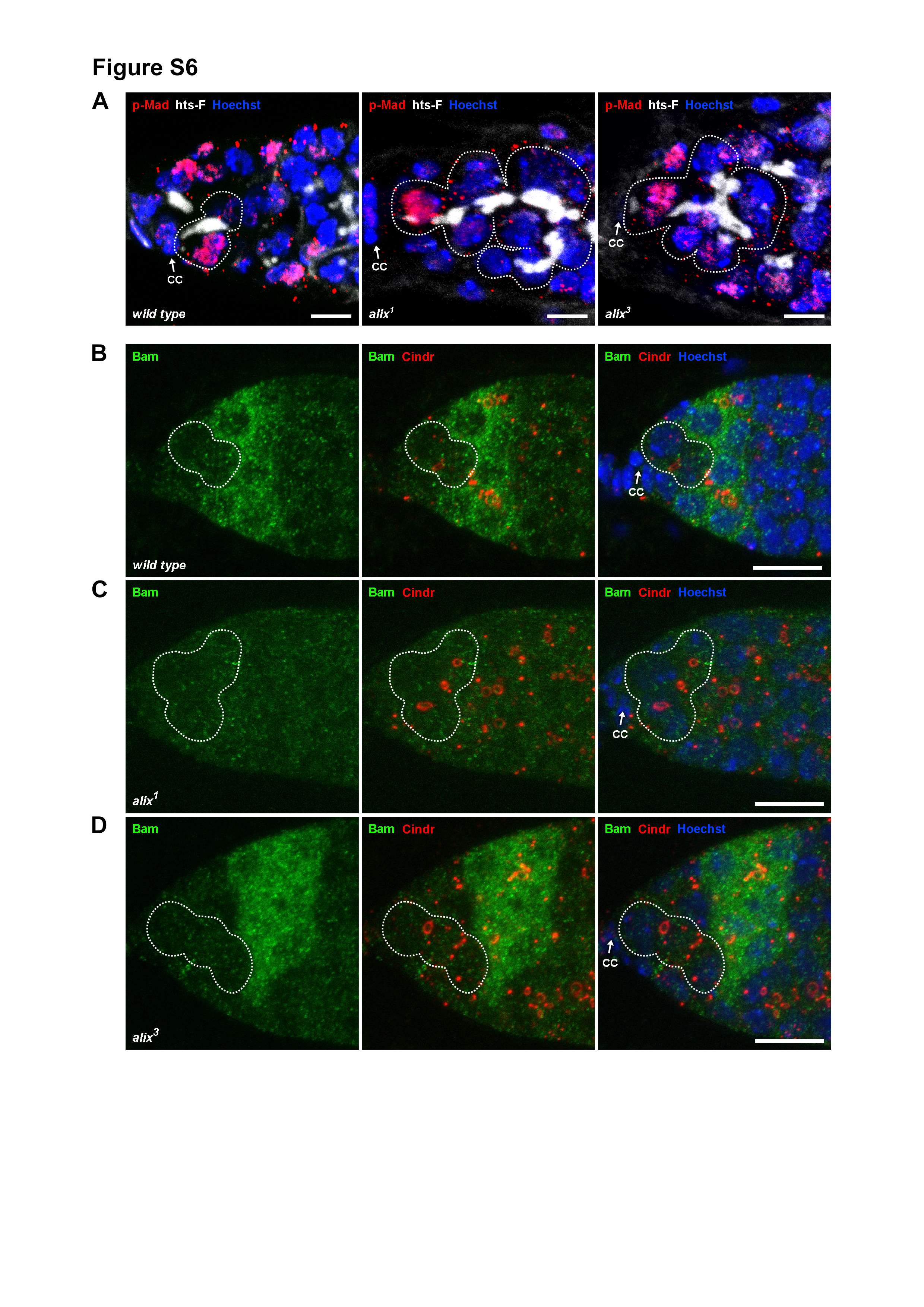

Supplement: S6 Fig — (A) Examples of wild type, alix1 and alix3 germaria stained for p-MAD (red) are shown. The wild type p-Mad-positive fGSC (red) is connected to a daughter cell CB via fusome (white). The alix1 and alix3 p-Mad-positive fGSCs (red) are connected chains of daughter cell via elongated fusomes. CC, cap cell. Scale bars represent 5μm. (B-D) Images showing Bam protein staining of wild type, alix1 and alix3 germaria. An fGSC-CB pair (B) or stem cysts (C-D) are outlined. Ovaries were stained with antibodies against Bam (green) and Cindr (red), and with Hoechst (blue). Scale bars represent 10 µm. (TIF) [file pgen.1004904.s006.tif]

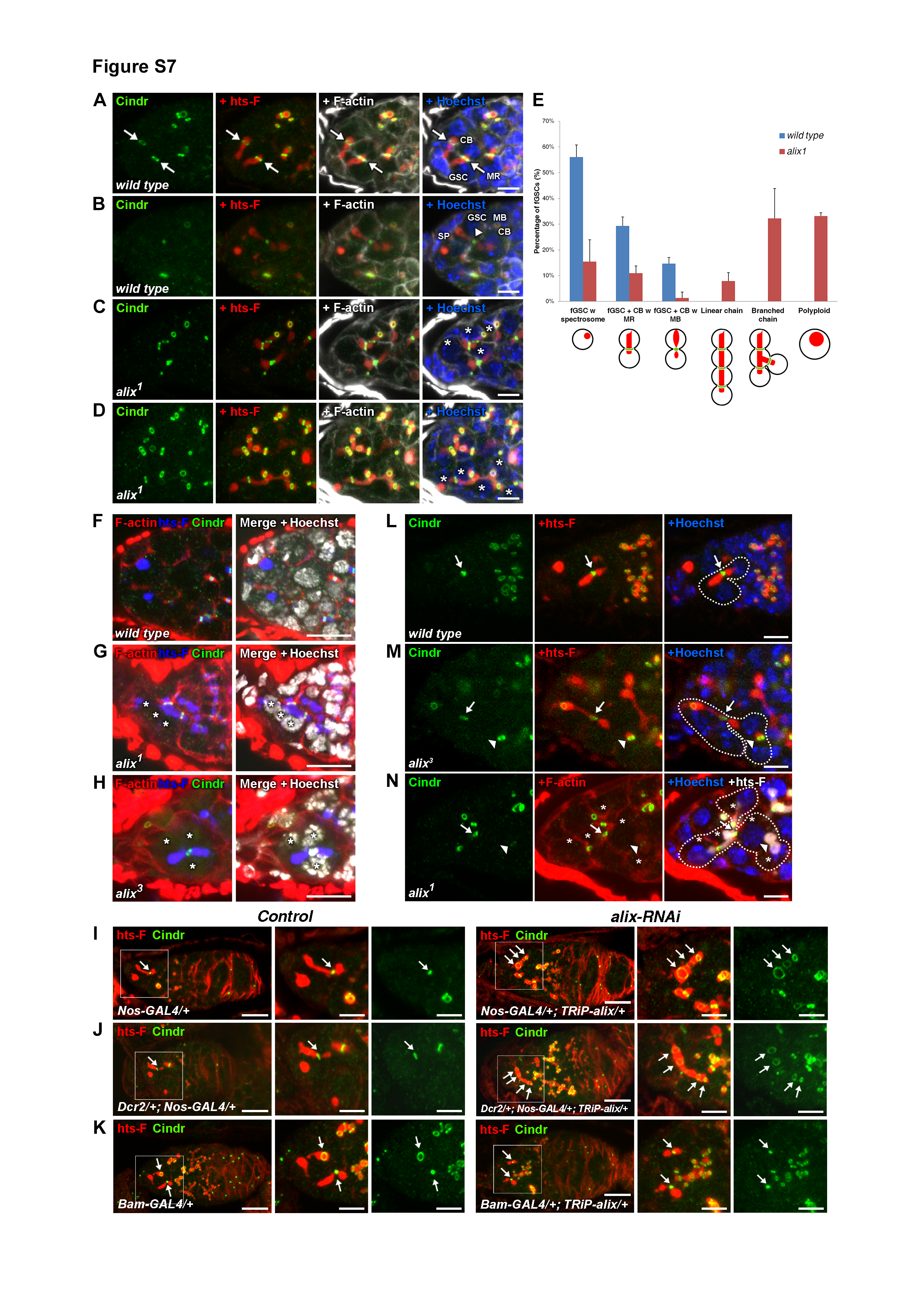

Supplement: S7 Fig — (A-B) Shown are examples wild type germaria with normal fGSC morphologies: (i) a single fGSCs with a spectrosome (red, SP), (ii) fGSC-CB pairs undergoing cytokinesis with midbody rings (MR, green, arrows) and fused fusomes (red) and (iii) an fGSC-CB pair in late cytokinesis with a midbody (MB, green, arrowhead) and fusome with exclamation point morphology (red). (C-D) alix1 mutant germaria show abnormal fGSC morphologies. Shown are fGSCs connected to more than one daughter cell in (iv) linear (C) or (v) branched (D) chains via midbody rings (green) and fusome (red). Cells interconnected are marked with asterisks. Ovaries in (A-D) were fixed and stained with antibodies against Cindr (green) and hts-F (red), with phalloidin to visualize F-actin (white) and with Hoechst (blue). Scale bars in (A-D) represent 5 µm. (E) Graph showing the average percentage of fGSCs with the indicated phenotypes from wild type and alix1 mutant flies. Wild type, three independent experiments, n = 110, 30 germaria; alix1, three independent experiments, n = 70, 29 germaria. The larger proportion of polyploid fGSCs in the alix1 compared to the alix3 mutant germaria might be explained by the fact that the alix1 mutant flies were older than the alix3 mutant females (7 days compared to 2–4 days old), allowing for more time for cleavage furrow regression. Data are presented as mean ± STD. See also S4 Table. (F) Wild-type germarium with normal fGSC morphologies. Shown are fGSCs with spectrosomes (hts-F, blue). (G-H) In alix1 (G) and alix3 (H) mutant germaria, bi- and multinucleate fGSCs can be detected. Nuclei of cells with more than one nucleus are marked with asterisks. Bi- and multi-nucleate phenotypes were mostly detected in flies older than four days and in some cases the midbody ring was still visible in the cell (H), indicating that it lost the connection to the plasma membrane. Ovaries in (F-H) were fixed and stained with antibodies against Cindr (green) and hts-F (blue), with rhodamine-pha [file pgen.1004904.s007.tif]

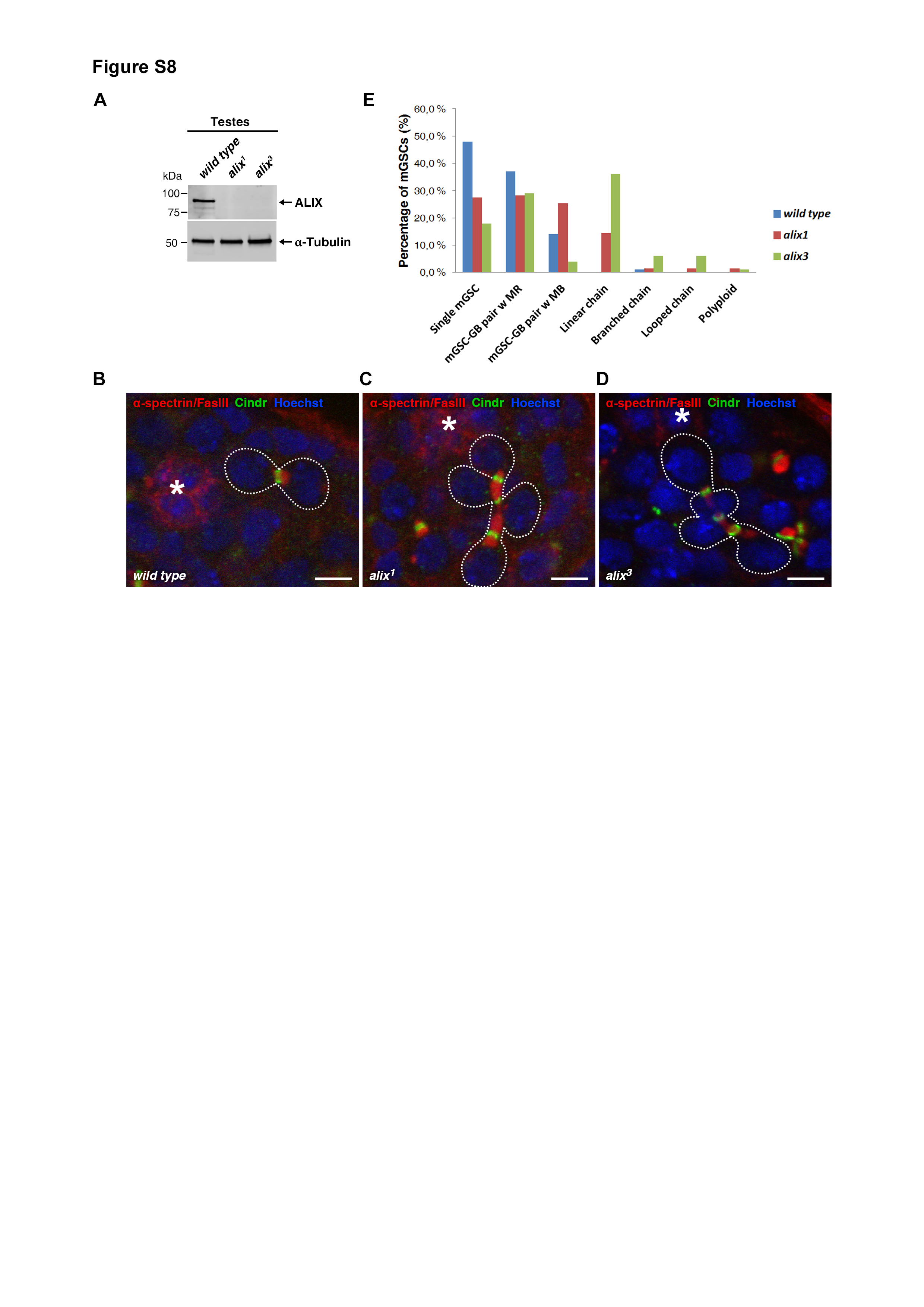

Supplement: S8 Fig — (A) Western blot showing the lack of ALIX protein alix1 and alix3 mutant testes. Equal protein loading is validated by the levels of α-tubulin. (B) Wild type testis tip with mGSC-gonialblast (GB) pair (outlined) in cytokinesis interconnected by an MR (Cindr, green) and fusome (α-spectrin, red). (C-D) Testis tips in alix1 and alix3 mutants with mGSCs connected to chains of daughter cells (outlined) via MRs (green) and fusome (red). Testes in (B-D) were fixed and stained with antibodies against Cindr (green), α-spectrin (red) and FasIII (red), and with Hoechst (blue). Hubs are indicated with asterisks. Scale bars represent 5 µm. (E) Graph showing the average percentage of mGSCs with the indicated phenotypes. Wild type, three independent experiments, n = 166 mGSCs, 17 testes; alix1, three independent experiments, n = 201 mGSCs, 17 testes; alix3, three independent experiments, n = 119 mGSCs, 17 testes. (TIF) [file pgen.1004904.s008.tif]

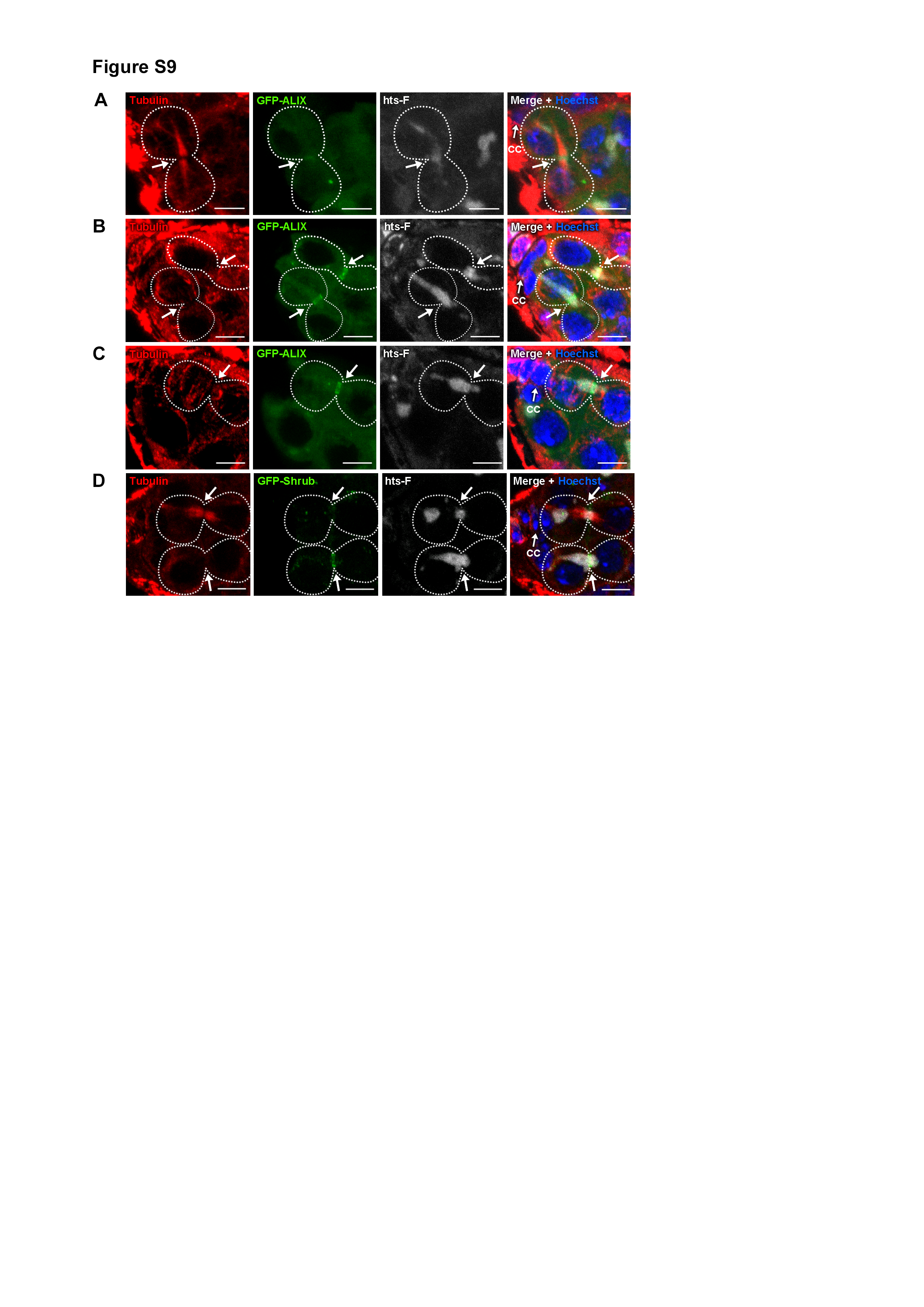

Supplement: S9 Fig — (A-D) Ovaries of 1–2 day-old UASp-GFP-ALIX/+; Nanos-GAL4/+ (A-C) or Nanos-GAL4, UASp-GFP-Shrub (D) flies were dissected, fixed and stained with anti-α-tubulin (red), GFP Booster (green), anti-hts-F (white) and Hoechst (blue). Arrows indicate localization of GFP-ALIX to fusome plugs (A), MRs in G1/S (B, bar-shaped fusome), S phase (C, dumbbell-shaped fusome) and G2 (B, fusing fusome). Intercellular bridge MTs are present early when the fusome has plug morphology. At this point only weak GFP-Shrub signal is detected and in G2 phase GFP-Shrub is detected at an MR (D). Scale bars represent 5 µm. (TIF) [file pgen.1004904.s009.tif]

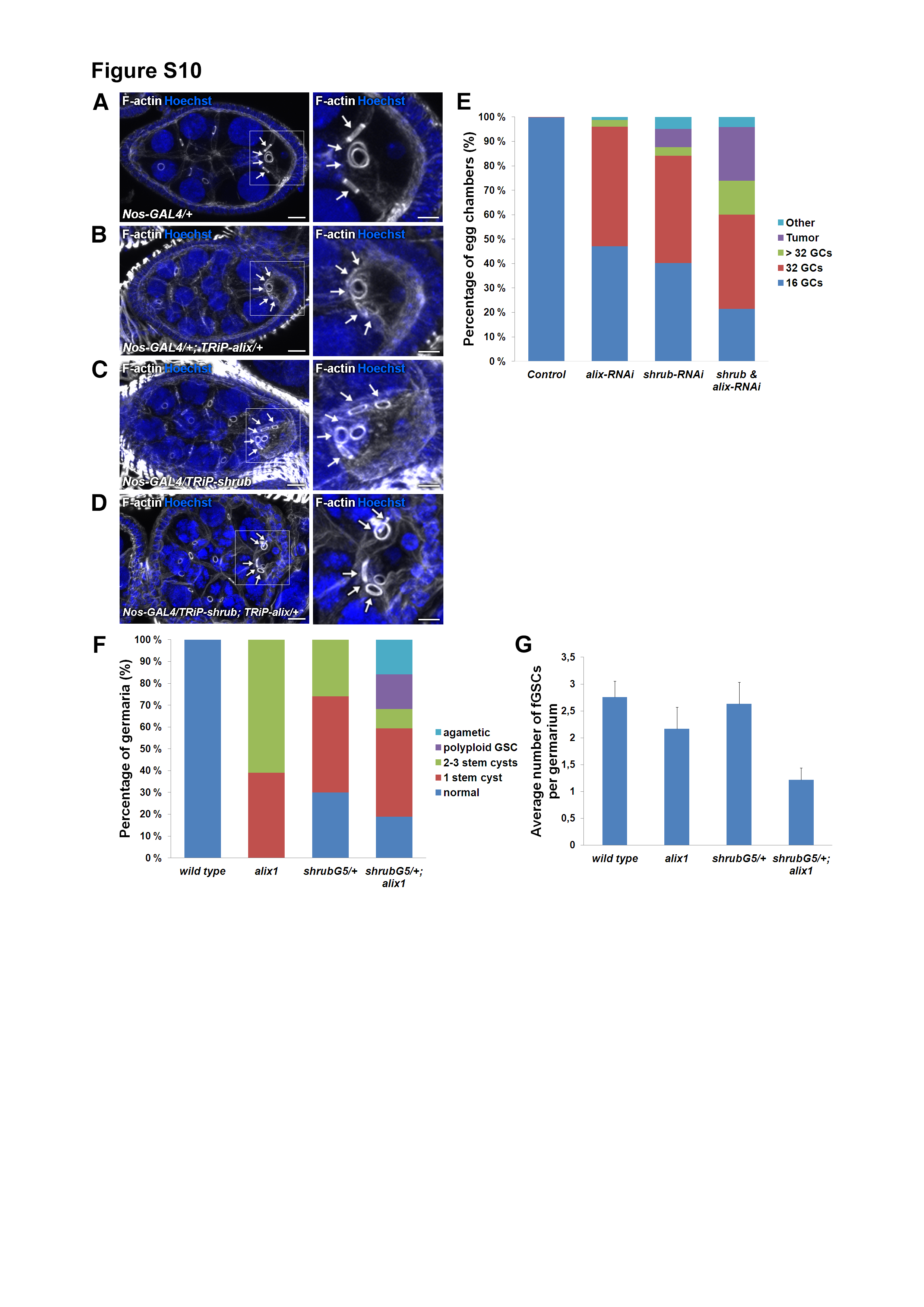

Supplement: S10 Fig — (A-D) Control EC with 4 RCs (arrows) to the oocyte (A) and alix-RNAi (B), shrub-RNAi (C) as well as shrub- and alix-RNAi (D) ECs with 5 RCs (arrows) to the oocyte. Ovaries were fixed and stained with phalloidin to visualize F-actin (white) and with Hoechst (blue). Scale bars represent 10 µm (left images) and 5 µm (right images). (E) Graph showing the average percentage of egg chambers (ECs) with 16, 32, more than 32 GCs, tumor phenotype and other phenotypes from control, alix-RNAi, shrub-RNAi, and shrub & alix-RNAi flies. Control, four independent experiments, n = 493 ECs; alix-RNAi, four independent experiments, n = 509 ECs; shrub-RNAi, four independent experiments, n = 231 ECs; shrub & alix-RNAi, 3 independent experiments, n = 215 ECs. (F) Graph showing the average percentages of germaria with the indicated phenotypes from wild type, alix1, shrubG5/+ and shrubG5/+; alix1 germaria. Wild type, four independent experiments, n = 29 germaria; alix1, three independent experiments, n = 23 germaria; shrubG5/+, three independent experiments, n = 27 germaria; shrubG5/+; alix1, three independent experiments, n = 33 germaria. (G) Graph showing the average number of stem cells per germarium from the wild type, alix1, shrubG5/+ and shrubG5/+; alix1 germaria in (F). Wild type, n = 80 fGSCs; alix1, n = 50 fGSCs; shrubG5/+, n = 70 fGSCs; shrubG5/+; alix1, n = 54 fGSCs. Data are presented as mean ± STD. (TIF) [file pgen.1004904.s010.tif]

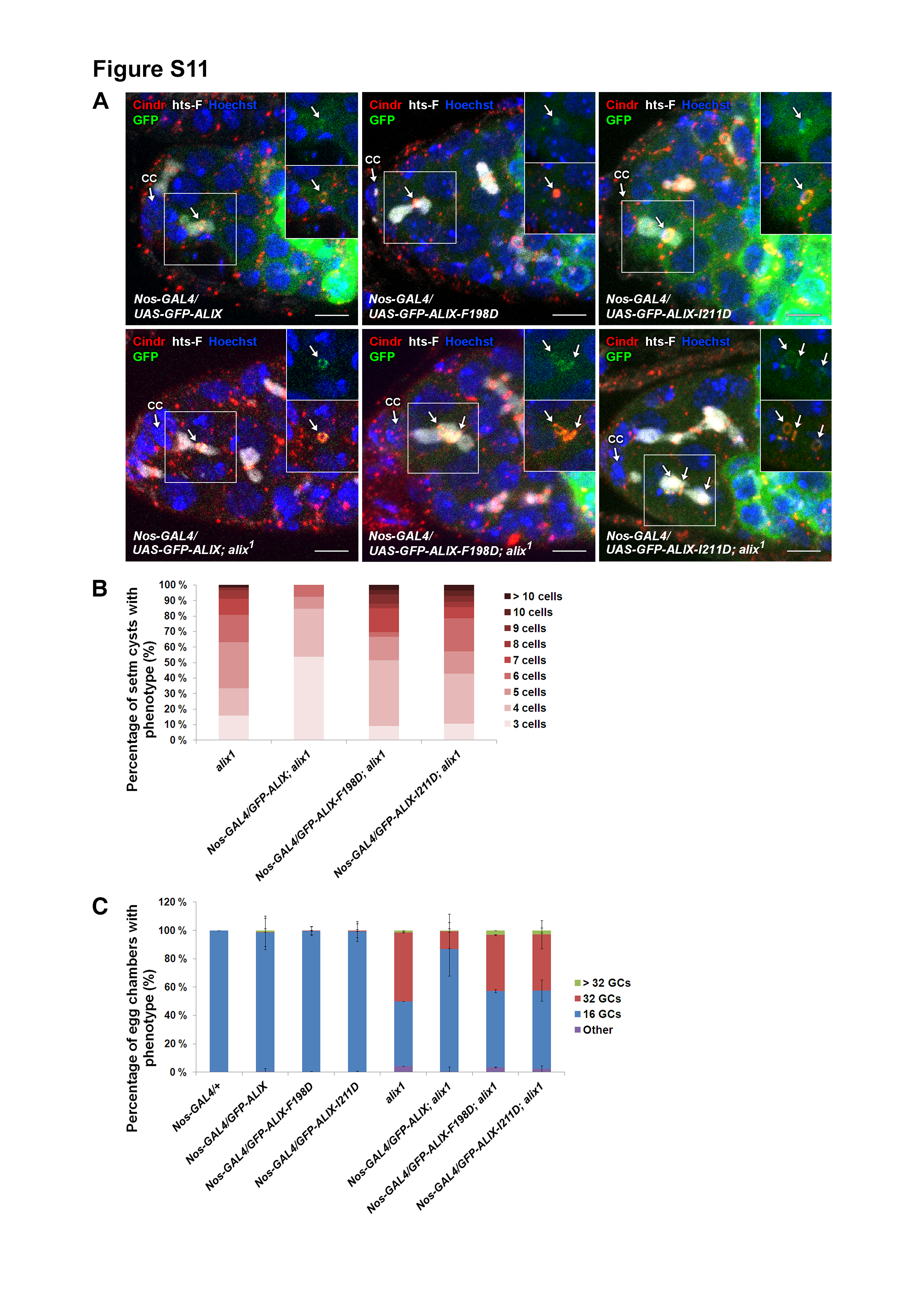

Supplement: S11 Fig — (A) Images showing the localization of wild type GFP-ALIX, GFP-ALIX-F198D or GFP-ALIX-I211D at MRs/MBs in fGSC-CB pairs or at MRs in stem cysts in germaria of the indicated genotypes. Ovaries were fixed and stained with antibodies against Cindr (red) and hts-F (white) and with Hoechst (blue). CC, cap cell. Scale bars represent 5 µm. (B) Graph showing the frequencies of stem cyst lengths for the indicated genotypes from the experiment in Fig. 7C. (C) Graph showing the average percentages of egg chambers with the indicated phenotypes in ovaries of females with the indicated genotypes from three independent experiments. Nanos-GAL4/+, n = 391 ECs; Nanos-GAL4/UASp-GFP-ALIX, 381 ECs; Nanos-GAL4/UASp-GFP-ALIX-F198D, 357 ECs; Nanos-GAL4/UASp-GFP-ALIX-I211D, 485 ECs; alix1, 508 ECs; Nanos-GAL4/UASp-GFP-ALIX; alix1, 392 ECs; Nanos-GAL4/UASp-GFP-ALIX-F198D; alix1, 500 ECs; Nanos-GAL4/UASp-GFP-ALIX-I211D; alix1, 378 ECs. Data are presented as mean ± STD. (TIF) [file pgen.1004904.s011.tif]
