# Supplementary material for: ALIX and ESCRT-III Coordinately Control Cytokinetic Abscission during Germline Stem Cell Division In Vivo
Source: PLoS Genet. 2015 Jan 30;11(1):e1004904. doi: 10.1371/journal.pgen.1004904 (PMC4312039; doi:10.1371/journal.pgen.1004904)
Supplement: S1 Table — (DOCX) [file pgen.1004904.s012.docx]

**Table S1. Percentages of follicle cells in *wild type*, *alix^1^* and *alix^3^* mutant egg chambers with one or more nuclei.**

| **Genotype** | **# of follicle cells** | **Percentage of follicle cells with phenotype** | | | |
| --- | --- | --- | --- | --- | --- |
|  |  | **1 nucleus** | **2 nuclei** | **3 nuclei** | **> 3 nuclei** |
| ***Wild type*** | 848 | 99,7 ± 0,2% | 0,3 ± 0,2% | 0,0 % | 0,0 % |
| ***alix^1^*** | 896 | 96,9 ± 1,0% | 2,8 ± 0,9% | 0,2 ± 0,4% | 0,0 % |
| ***alix^3^*** | 802 | 98,7 ± 1,1% | 1,3 ± 1,1 % | 0,0 % | 0,0 % |

Egg chamber (EC) stages until stage 8 were analyzed. *Wild type*, n = 848 follicle cells (FCs) from 24 ECs; *alix^1^*, n = 896 FCs from 28 ECs; *alix^3^*, n = 802 FCs from 27 ECs. Data are based on three independent experiments and presented as mean ± STD.
