# Supplementary material for: ALIX and ESCRT-III Coordinately Control Cytokinetic Abscission during Germline Stem Cell Division In Vivo
Source: PLoS Genet. 2015 Jan 30;11(1):e1004904. doi: 10.1371/journal.pgen.1004904 (PMC4312039; doi:10.1371/journal.pgen.1004904)
Supplement: S2 Table — (DOCX) [file pgen.1004904.s013.docx]

**Table S2. Percentage of GFP-positive control and *alix^3^* mutant follicle cells with one or more nuclei.**

| **Genotype** | **Egg chamber stage** | **# of GFP-positive follicle cells** | **Percentage of GFP-positive follicle cells with phenotype** | | | | |
| --- | --- | --- | --- | --- | --- | --- | --- |
|  |  |  | **1 nucleus** | **2 nuclei** | **3 nuclei** | **4 nuclei** | **> 4 nuclei** |
| ***Control*** | 10 | 146 | 99% | 1% | 0% | 0% | 0% |
|  | 14 | 156 | 99% | 1% | 0% | 0% | 0% |
| ***alix^3^*** | 10 | 350 | 97% | 3% | 0% | 0% | 0% |
|  | 14 | 252 | 72% | 23% | 3% | 2% | 0% |
