# Supplementary material for: ALIX and ESCRT-III Coordinately Control Cytokinetic Abscission during Germline Stem Cell Division In Vivo
Source: PLoS Genet. 2015 Jan 30;11(1):e1004904. doi: 10.1371/journal.pgen.1004904 (PMC4312039; doi:10.1371/journal.pgen.1004904)
Supplement: S4 Table — (DOCX) [file pgen.1004904.s015.docx]

**Table S4. Number of *wild type* and *alix^1^* mutant germaria with normal versus abnormal fGSC phenotypes.**

| **Genotype** | **Experiment** | **Number of germaria with fGSC phenotype** | | **Total number of germaria** |
| --- | --- | --- | --- | --- |
|  |  | **Normal*** | **Abnormal**** |  |
| ***wild type*** | **1** | 10 | 0 | 10 |
|  | **2** | 10 | 0 | 10 |
|  | **3** | 10 | 0 | 10 |
| ***alix^1^*** | **1** | 1 | 8 | 9 |
|  | **2** | 1 | 9 | 10 |
|  | **3** | 0 | 10 | 10 |

* Germaria with normal fGSC phenotypes are defined as those with the fGSC phenotypes defined as normal in the main text ((i) fGSC with spectrosome, (ii) fGSC-CB pair with MR and (iii) fGSC-CB pair with MB).

** Germaria with abnormal fGSC phenotypes are defined based on the presence of at least one fGSC with an abnormal fGSC phenotype as defined in the main text and Materials and Methods ((iv) linear chain, (v) branched chain or (vi) polyploidy) and quantified from the z-stacks in the three experiments in Figure S7E. Abscission defects appeared in the majority of *alix^1^* mutant germaria, and never in *wild type* , which was a systematically significant difference between *wild type* and either *alix* mutant in each experiment (p<0.0001, Fisher’s exact test).
