# Supplementary material for: Correction: ALIX and ESCRT-III Coordinately Control Cytokinetic Abscission during Germline Stem Cell Division In Vivo
Source: PLoS Genet. 2015 Jun 22;11(6):e1005314. doi: 10.1371/journal.pgen.1005314 (PMC4476838; doi:10.1371/journal.pgen.1005314)
Supplement: S2 File — (PDF) [file pgen.1005314.s002.pdf]

PLOS | GENETICS

RESEARCH ARTICLE

# ALIX and ESCRT-III Coordinately Control Cytokinetic Abscission during Germline Stem Cell Division *In Vivo*

**Åsmund H. Eikenes[1,2‡], Lene Malerød[1,2‡], Anette Lie Christensen[1,2], Chloé B. Steen[1,2], Juliette Mathieu[3,4], Ioannis P. Nezis[5], Knut Liestøl[2,6], Jean-René Huynh[3,4], Harald Stenmark[1,2], Kaisa Haglund[1,2]***

**1** Department of Biochemistry, Institute for Cancer Research, Oslo University Hospital, Oslo, Norway, **2** Centre for Cancer Biomedicine, Faculty of Medicine, University of Oslo, Oslo, Norway, **3** Department of Genetics and Developmental Biology, Institut Curie, Paris, France, **4** CNRS UMR3215, Inserm U934 F-75248, Paris, France, **5** School of Life Sciences, University of Warwick, Coventry, United Kingdom **6** Department of Informatics, University of Oslo, Oslo, Norway

‡ These authors contributed equally to this work.
* Kaisa.Haglund@rr-research.no

CrossMark
click for updates

OPEN ACCESS

**Citation:** Eikenes ÅH, Malerød L, Christensen AL, Steen CB, Mathieu J, Nezis IP, et al. (2015) ALIX and ESCRT-III Coordinately Control Cytokinetic Abscission during Germline Stem Cell Division *In Vivo*. PLoS Genet 11(1): e1004904. doi:10.1371/journal.pgen.1004904

**Editor:** Gregory P. Copenhaver, The University of North Carolina at Chapel Hill, UNITED STATES

**Received:** June 12, 2014

**Accepted:** November 18, 2014

**Published:** January 30, 2015

**Copyright:** © 2015 Eikenes et al. This is an open access article distributed under the terms of the Creative Commons Attribution License, which permits unrestricted use, distribution, and reproduction in any medium, provided the original author and source are credited.

**Data Availability Statement:** All relevant data are within the paper and its Supporting Information files.

**Funding:** ÅHE is a PhD student of The Norwegian Cancer Society, project number 605009 (https://kreftforeningen.no/). HS acknowledges support by an Advanced Grant from the European Research Council, project number 233146 (http://erc.europa.eu/). KH acknowledges a career researcher grant from the South-Eastern Norway Regional Health Authority, project number 2012054 (http://www.helse-sorost.no). LM and ALC are supported by this grant. KH also acknowledges funding from the Research Council of

## Abstract

Abscission is the final step of cytokinesis that involves the cleavage of the intercellular bridge connecting the two daughter cells. Recent studies have given novel insight into the spatiotemporal regulation and molecular mechanisms controlling abscission in cultured yeast and human cells. The mechanisms of abscission in living metazoan tissues are however not well understood. Here we show that ALIX and the ESCRT-III component Shrub are required for completion of abscission during Drosophila female germline stem cell (fGSC) division. Loss of ALIX or Shrub function in fGSCs leads to delayed abscission and the consequent formation of stem cysts in which chains of daughter cells remain interconnected to the fGSC via midbody rings and fusome. We demonstrate that ALIX and Shrub interact and that they co-localize at midbody rings and midbodies during cytokinetic abscission in fGSCs. Mechanistically, we show that the direct interaction between ALIX and Shrub is required to ensure cytokinesis completion with normal kinetics in fGSCs. We conclude that ALIX and ESCRT-III coordinately control abscission in *Drosophila* fGSCs and that their complex formation is required for accurate abscission timing in GSCs *in vivo*.

## Author Summary

Cytokinesis, the final step of cell division, concludes with a process termed abscission, during which the two daughter cells physically separate. In spite of their importance, the molecular machineries controlling abscission are poorly characterized especially in the context of living metazoan tissues. Here we provide molecular insight into the mechanism of abscission using the fruit fly *Drosophila melanogaster* as a model organism. We show

Norway, project number 191569 (http://www.forskningsradet.no) and an EMBO Short Term Fellowship. This work was partly supported by the Research Council of Norway through its Centres of Excellence funding scheme, project number 179571. The funders had no role in study design, data collection and analysis, decision to publish, or preparation of the manuscript.

**Competing Interests:** The authors have declared that no competing interests exist.

that the scaffold protein ALIX and the ESCRT-III component Shrub are required for completion of abscission in *Drosophila* female germline stem cells (fGSCs). ESCRT-III has been implicated in topologically similar membrane scission events as abscission, namely intraluminal vesicle formation at endosomes and virus budding. Here we demonstrate that ALIX and Shrub co-localize and interact to promote abscission with correct timing in *Drosophila* fGSCs. We thus show that ALIX and ESCRT-III coordinately control abscission in *Drosophila* fGSCs cells and report an evolutionarily conserved function of the ALIX/ESCRT-III pathway during cytokinesis in a multi-cellular organism.

## Introduction

Cytokinesis is the final step of cell division that leads to the physical separation of the two daughter cells. It is tightly controlled in space and time and proceeds in multiple steps via sequential specification of the cleavage plane, assembly and constriction of the actomyosin-based contractile ring (CR), formation of a thin intercellular bridge and finally abscission that separates the two daughter cells [1–8]. Studies in a variety of model organisms and systems have elucidated key machineries and signals governing early events of cytokinesis [1–6]. However, the mechanisms of the final abscission step of cytokinesis are less understood, especially *in vivo* in the context of different cell types in a multi-cellular organism [2, 4, 5].

During the recent years key insights into the molecular mechanisms and spatiotemporal control of abscission have been gained using a combination of advanced molecular biological and imaging technologies [4, 7, 9–15]. At late stages of cytokinesis the spindle midzone transforms to densely packed anti-parallel microtubules (MTs) that make up the midbody (MB) and the CR transforms into the midbody ring (MR, diameter of ~1–2 μm) [4, 10, 16, 17]. The MR is located at the site of MT overlap and retains several CR components including Anillin, septins (Septins 1, 2 and Peanut in *Drosophila melanogaster*), myosin-II, Citron kinase (Sticky in *Drosophila*) and RhoA (Rho1 in *Drosophila*) and eventually also acquires the centralspindlin component MKLP1 (Pavarotti in *Drosophila*) [4, 16, 18, 19]. In *C. elegans* embryos the MR plays an important role in scaffolding the abscission machinery even in the absence of MB MTs [20].

Studies in human cell lines, predominantly in HeLa and MDCK cells, have shown that components of the endosomal sorting complex required for transport (ESCRT) machinery and associated proteins play important roles in mediating abscission [4, 7, 9–15]. Abscission occurs at the thin membrane neck that forms at the constriction zone located adjacent to the MR [9, 10, 17]. An important signal for initiation of abscission is the degradation of the mitotic kinase PLK1 (Polo-like kinase 1) that triggers the targeting of CEP55 (centrosomal protein of 55 kDa) to the MR [21]. CEP55 interacts directly with GPP(3x)Y motifs in the ESCRT-associated protein ALIX (ALG-2-interacting protein X) and in the ESCRT-I component TSG101, thereby recruiting them to the MR [13–15, 22]. ALIX and TSG101 in turn recruit the ESCRT-III component CHMP4B, which is followed by ESCRT-III polymerization into helical filaments that spiral/slide to the site of abscission [9, 11, 13–15, 23]. The VPS4 ATPase is thought to promote ESCRT-III redistribution toward the abscission site [23]. Prior to abscission ESCRT-III/CHMP1B recruits Spastin that mediates MT depolymerization at the abscission site [9, 10, 24]. ESCRT-III then facilitates membrane scission of the thin membrane neck, thereby mediating abscission [9, 10].

Cytokinesis is tightly controlled by the activation and inactivation of mitotic kinases at several steps to ensure its faithful spatiotemporal progression [7, 8]. Cytokinesis conventionally

proceeds to completion via abscission, but is differentially controlled depending on the cell type during the development of metazoan tissues. For example, germ cells in species ranging from insects to humans undergo incomplete cytokinesis leading to the formation of germline cysts in which cells are interconnected via stable intercellular bridges [25–27]. How cytokinesis is modified to achieve different abscission timing in different cell types is not well understood, but molecular understanding of the regulation of the abscission machinery has started giving some mechanistic insight [25, 26, 28–30].

The *Drosophila* female germline represents a powerful system to address mechanisms controlling cytokinesis and abscission *in vivo* [29, 31]. Each *Drosophila* female germline stem cell (fGSC) divides asymmetrically with complete cytokinesis to give rise to another fGSC and a daughter cell cystoblast (CB) [31–33]. Cytokinesis during fGSC division is delayed so that abscission takes place during the G2 phase of the following cell cycle (about 24 hours later) [31]. The CB in turn undergoes four mitotic divisions with incomplete cytokinesis giving rise to a 16-cell cyst in which the cells remain interconnected by stable intercellular bridges called ring canals (RCs) [27, 32]. One of the 16 cells with four RCs will become specified as the oocyte and the cyst becomes encapsulated by a single layer follicle cell epithelium to form an egg chamber [34, 35]. *Drosophila* male GSCs (mGSCs) also divide asymmetrically with complete cytokinesis to give rise to another mGSC and a daughter cell gonialblast (GB) [33, 36, 37]. Anillin, Pavarotti, Cindr, Cyclin B and Orbit are known factors localizing at RCs/MRs and/or MBs during complete cytokinesis in fGSCs and/or mGSCs [29, 31, 36, 38–43]. Mathieu *et al*. recently reported that Aurora B delays abscission and that Cyclin B promotes abscission in *Drosophila* germ cells and that mutual inhibitions between Aurora B and Cyclin B/Cdk-1 control the timing of abscission in *Drosophila* fGSCs and germline cysts [29]. However, little is known about further molecular mechanisms controlling cytokinesis and abscission in *Drosophila* fGSCs.

Here we characterize the roles of ALIX and the ESCRT-III component Shrub during cytokinesis in *Drosophila* fGSCs. We find that ALIX and Shrub are required for completion of abscission in fGSCs, that they co-localize during this process and that their direct interaction is required for abscission with normal kinetics. We thus show that a complex between ALIX and Shrub is required for abscission in fGSCs and provide evidence of an evolutionarily conserved functional role of the ALIX/ESCRT-III pathway in mediating cytokinetic abscission in the context of a multi-cellular organism.

## Results

### ALIX localizes at the midbody during cytokinesis in *Drosophila* cells

The ESCRT-associated scaffold protein ALIX promotes cytokinetic abscission in human cultured cells [13–15]. We were interested to characterize the role of ALIX in cytokinesis *in vivo* using *Drosophila melanogaster* as a model because of its power for elucidation of mechanisms of cytokinesis and abscission in different cell types in a developing organism [2, 5, 6, 29]. We first raised an antibody against *Drosophila* ALIX (*CG12876*) (Fig. 1A) and examined its subcellular localization during S2 cell division. During meta-, ana- and early telophase ALIX localized at centrosomes (Fig. 1B-E), where it co-localized with Centrosomin (S1A-S1C Fig.). ALIX localization at centrosomes has been detected in human cultured cells in interphase [15], but to our knowledge ALIX localization at centrosomes during different phases of mitosis has not previously been shown. Strikingly, at mid telophase a fraction of ALIX re-localized from the spindle poles to two pools within the intercellular bridge on each side of the MR/dark zone (Figs. 1F and S1D). Finally, ALIX localized to the central region of the intercellular bridge during late telophase/cytokinesis (Figs. 1G and S1E). Here it appeared to localize to the MR because it formed a ring-like structure around the MTs of the MB (Fig. 1G) at the dark zone

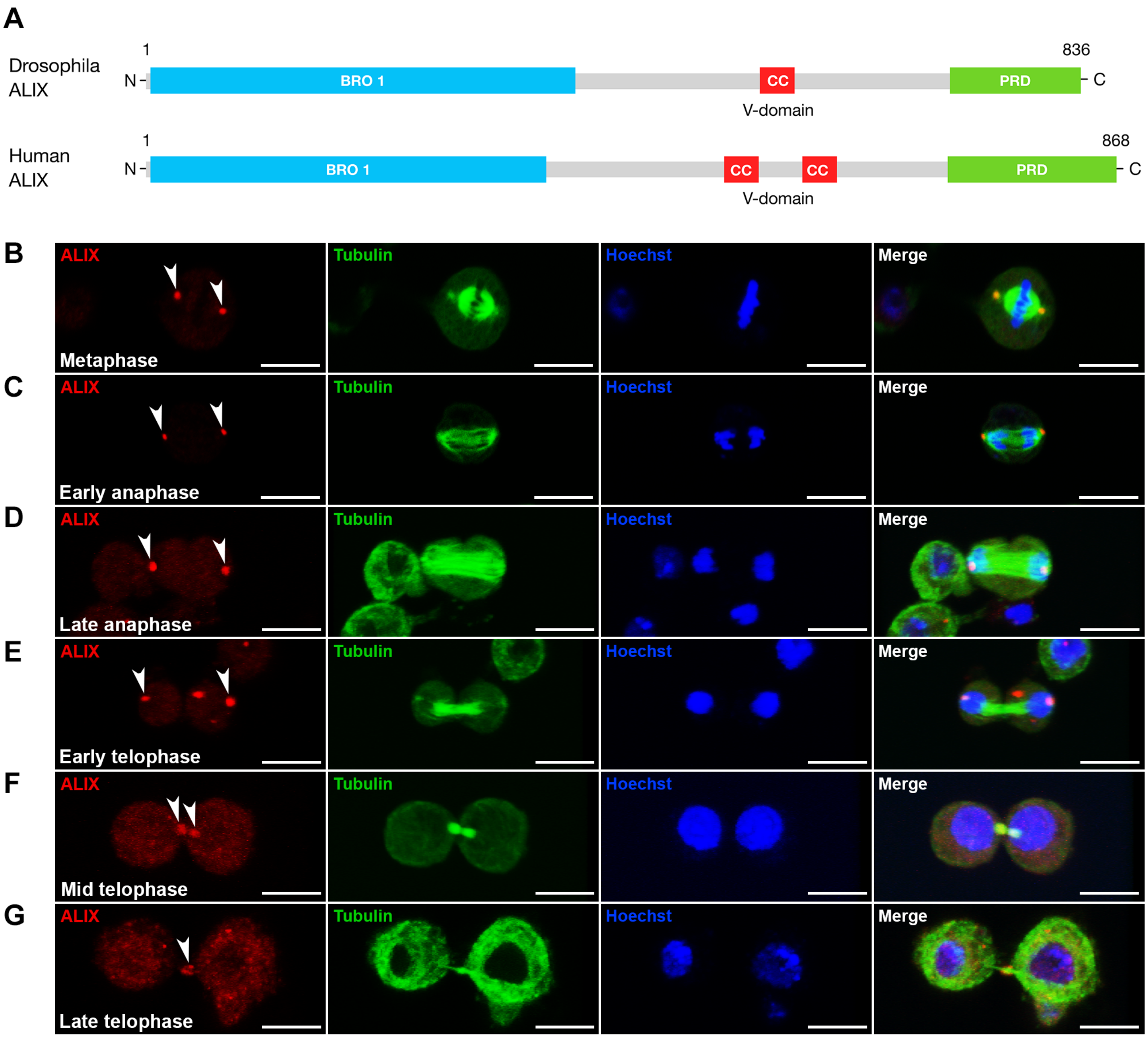


**Figure 1. ALIX localizes at the midbody ring during cytokinesis in *Drosophila* S2 cells. (A)** Schematic of *Drosophila* and human ALIX domain structures. The *Drosophila* ALIX protein shows ~60% homology to human ALIX and contains an N-terminal Bro1 domain (BRO1), a central coiled-coil (CC) and a C-terminal proline-rich domain (PRD). **(B-E)** ALIX localizes at centrosomes in (B) metaphase, (C) early anaphase, (D) late anaphase and (E) early telophase. **(F)** In mid telophase, ALIX localizes at the ICB in two pools that overlap with α-tubulin on each side of the central region of the MB. **(G)** In late telophase/cytokinesis, ALIX localizes at the MR. In (B-G), S2 cells stably expressing GFP-α-tubulin (green) were fixed and stained with a guinea pig anti-ALIX antibody (red), and with Hoechst (blue). Scale bars represent 5 μm. See also S1 Fig.

doi:10.1371/journal.pgen.1004904.g001

(S1E Fig.). The pre-immune serum neither stained centrosomes, nor the intercellular bridge or MR (S1A-S1E Fig.). This spatiotemporal redistribution from centrosomes to the MR suggested a possible role for ALIX in cytokinesis in *Drosophila* cells.

## Loss of ALIX gives rise to egg chambers with 32 germ cells during *Drosophila* oogenesis

We further addressed the role of *Drosophila* ALIX in cytokinesis *in vivo* by analyzing two different *alix* mutant alleles, $alix^1$ and $alix^3$ (Fig. 2A). ALIX is highly expressed in *Drosophila*

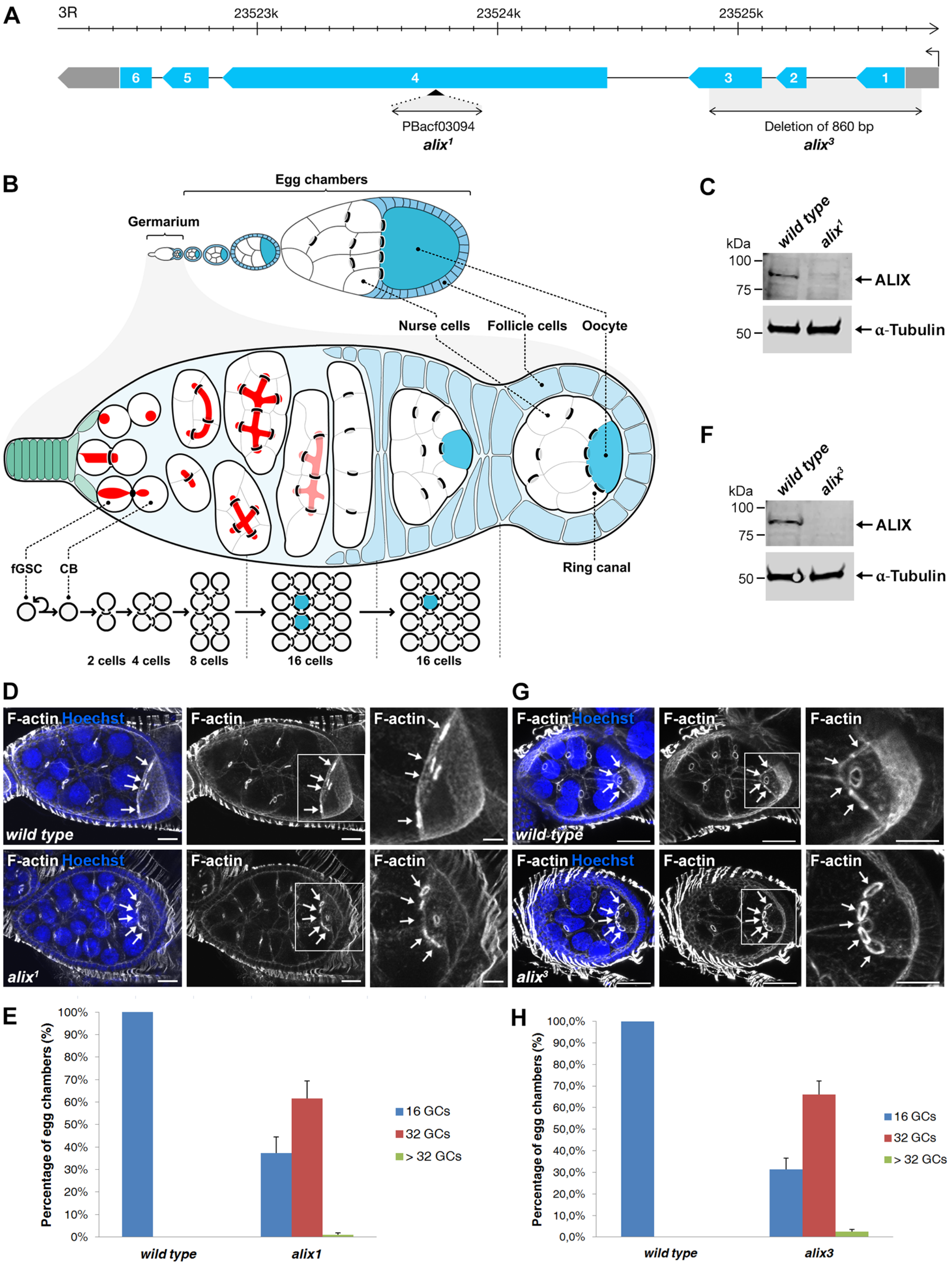


**Figure 2. Loss of ALIX gives rise to egg chambers with increased number of germ cells during *Drosophila* oogenesis. (A)** Schematic of the *Drosophila alix* gene locus and the *alix*[1] and *alix*[3] alleles. The *alix* gene (*CG12876*) is located on 3R, band 98B1, and is encoded by six exons. **(B)** Overview of *Drosophila* oogenesis, female germline stem cell (fGSC) and germ cell divisions. Each fGSC in the stem cell niche in the anterior tip of the germarium divides with complete cytokinesis to give rise to another fGSC and a daughter cell, a cystoblast (CB). Cytokinetic abscission occurs as the MR closes to form an MB and the fusome (red) is cut in two unequal parts. The CB leaves the niche and undergoes four mitotic divisions with incomplete cytokinesis, giving rise to a

16-cell cyst in which the cells are interconnected by ring canals (RCs). One of the cells with four RCs will be specified as the oocyte. The 16-cell cyst becomes encapsulated by follicle cells to form an egg chamber, each of which will undergo 14 developmental stages to form an egg. **(C, F)** Western blots showing the lack of ALIX protein in (C) *alix*$^1$ and (F) *alix*$^3$ mutant ovaries, respectively. α-tubulin was used as a loading control. **(D, G)** *alix*$^1$ (D) and *alix*$^3$ (G) mutant egg chambers (ECs) frequently contain 32 germ cells (GCs). Upper panels: *Wild type* ECs with four RCs (arrows) to the oocyte. Lower panels: (D) *alix*$^1$ and (G) *alix*$^3$ mutant ECs with five RCs (arrows) to the oocyte. Ovaries were fixed and stained to visualize F-actin (white) and nuclei (blue). Scale bars represent 20 μm (left and middle images in each panel) and 10 μm (right images in each panel). **(E, H)** Graphs showing the average percentage of ECs with 16, 32 or more GCs from three independent experiments from *wild type* and *alix*$^1$ or *alix*$^3$ mutant flies, respectively. (E) *Wild type*, n = 548 ECs; *alix*$^1$, n = 273 ECs. (H) *Wild type*, n = 222 ECs; *alix*$^3$, n = 228 ECs. Data are based on three independent experiments and presented as mean ± STD in both (E) and (H). See also S3 and S4 Figs.

doi:10.1371/journal.pgen.1004904.g002

embryos, larvae, pupae, adult females and males, as well as in ovaries and testes (S2A Fig.). Interestingly, homozygous mutant offspring of both the *alix*$^1$ and *alix*$^3$ mutants could survive to adulthood (even though they clearly lack the full-length ALIX protein) (S2B Fig. and see below) and we detected none or only minor bi-nucleation clearly attributed to cytokinesis failure in the somatic cell types we analyzed (S2C-S2F Fig. and S1–S2 Tables). Fertility tests of *alix*$^1$ mutant flies however revealed that both female and male fertility was reduced (S3A–S3B Fig.). In particular female fertility was severely compromised, manifested by very low egg lay and hatch rates (S3A–S3B Fig.). We therefore asked whether oogenesis of *alix* mutant flies might be altered. *Wild type* egg chambers contain 16 germ cells and an oocyte with 4 RCs (Fig. 2B, 2D, 2G, 2E and 2H). Curiously, egg chambers in ovaries of both *alix*$^1$ and *alix*$^3$ mutant females lacking full-length ALIX (Fig. 2C and 2F) often contained exactly 32 germ cells and an oocyte with 5 RCs (Fig. 2D and 2G). Quantifying the egg chamber phenotypes of *alix*$^1$ and *alix*$^3$ mutant ovaries revealed that about 60% of the egg chambers in both alleles contained 32 germ cells (Fig. 2E and 2H). We also detected low percentages of egg chambers with more than 32 germ cells in both *alix* mutant alleles (Fig. 2E and 2H).

We next analyzed whether the increased germ cell number in egg chambers was specifically due to loss of *alix* gene function. Firstly, *alix*$^1$ and *alix*$^3$ alleles combined either with two different deficiencies lacking the *alix* gene or with each other gave rise to 50–60% of egg chambers with 32 germ cells, similar to homozygous *alix*$^1$ and *alix*$^3$ mutants (S3C-S3E Fig.). Secondly, two genomic rescue lines containing the full *alix* gene locus rescued the 32-germ cell phenotype of both the *alix*$^1$ and *alix*$^3$ alleles (S4A–S4F Fig.). Finally, RNAi-mediated gene silencing of *alix* specifically in female germ cells using the maternal triple *MTD-GAL4* driver [44–46] resulted in about 50% of egg chambers with 32 or more germ cells (S4G-S4I Fig.) showing that absence of ALIX specifically in germ cells causes the 32-germ cell phenotype. We conclude that loss of ALIX function in the *Drosophila* female germline causes the formation of a high frequency of egg chambers with 32 or more germ cells.

## Loss of ALIX results in the formation of stem cysts in the *Drosophila* female germline

Egg chambers with 32 germ cells may arise via encapsulation of two 16-cell cysts by the follicle cell epithelium, an extra round of mitosis in germline cysts or a delay in abscission in fGSCs [29, 32, 35, 47, 48]. The fact that the egg chambers with 32 germ cells contained one oocyte with 5 RCs excluded that they arose via defective encapsulation of two 16-cell cysts. We further discriminated between the two latter mechanisms by performing RNAi-mediated gene silencing of *alix* specifically in the germline using either *Nanos-GAL4* (expresses in all germ cells; fGSCs, CBs and 2–16-cell cysts) or *Bam-GAL4* (expresses in CBs to 8-cell cysts, but not in fGSCs) to test whether the phenotype originated from fGSCs or cell autonomously in germline cysts. Interestingly, *alix*-RNAi using *Nanos-GAL4* (*Nanos*-GAL4 or *UAS-Dicer; Nanos-GAL4*) gave rise to 40–60% egg chambers with 32 germ cells, whereas *alix* depletion using *Bam-GAL4*

resulted in normal egg chambers with 16 germ cells only (S5A-S5D Fig.). These data linked *alix* depletion in fGSCs to the formation of egg chambers with 32 germ cells and suggested that they did not arise from an extra round of mitosis of germline cysts. This thus indicated a role for ALIX in abscission in fGSCs in agreement with recent work showing that a delay in abscission in fGSCs can give rise to the formation of stem cysts in which the fGSC is connected to several daughter cells [29]. If abscission eventually takes place, a 2-cell cysts may pinch off and subsequently undergo four rounds of mitosis, giving rise to a 32-cell cyst [29]. We thus investigated whether or not we could detect stem cysts following loss of ALIX function.

Stem cysts are characterized by their elongated fusomes, their weak Nanos expression as in stem cells, their lack of expression of the cyst differentiation factor Bam and that the cell in direct contact with the stem cell niche is positive for p-Mad [29]. The cells within the stem cysts are moreover found to divide synchronously [29]. Importantly, $alix^1$ and $alix^3$ germaria as well as germaria with *alix*-RNAi in fGSCs displayed chains of weakly Nanos-positive germ cells interconnected by elongated fusomes in which the most anterior cell was in contact with the cap cells in the stem cell niche of the germarium (Figs. 3A-C and S5E-S5H). The cell in contact with the cap cells in such *alix*-deficient cysts was moreover p-Mad-positive (S6A Fig.) and the cysts were Bam-negative (S6B-S6D Fig.). We also detected synchronously dividing cells in the anterior tip of *alix*-deficient germaria (Fig. 3I). Taken together, these characteristics defined the *alix*-deficient cysts as stem cysts and indicated a role for ALIX in abscission in fGSCs.

## ALIX promotes abscission in *Drosophila* female germline stem cells

Each fGSC divides with complete cytokinesis giving rise to another stem cell and a daughter cell CB [31, 32] (Fig. 2B). fGSC cytokinesis progression can be monitored using markers for the fusome and RCs (hereafter referred to as MRs) [31, 32, 49, 50]. To determine the nature and frequency of the abscission defects upon loss of ALIX function we quantified fGSC morphologies in *wild type*, $alix^1$ and $alix^3$ germaria using markers for the fusome (hts-F), MRs/MBs (Cindr) [38] and nuclei (Figs. 3D-G and S7A-S7H). We categorized fGSC phenotypes as indicated in Fig. 3H (and as illustrated in S7E Fig.). *Wild type* fGSCs displayed only normal phenotypes: ~50% fGSCs with a spectrosome, ~40% fGSC-CB pairs with an MR and ~10% fGSC-CB pairs with an MB (Figs. 3D-E, 3H, S7A-S7B and S7E). These frequencies of different fGSC cell cycle stages are consistent with previous reports [49, 51]. $alix^3$ and $alix^1$ mutant germaria contained smaller fractions of fGSCs with a spectrosome (~15% for both mutants), fGSC-CB pairs with an MR (~25% in $alix^3$ and ~10% in $alix^1$) and fGSC-CB pairs with an MB (0% in $alix^3$ and ~1% in $alix^1$) compared to *wild type* (Figs. 3H and S7E). Importantly, more than half of the *alix* mutant fGSCs showed abscission defects: linear chains (~20% in $alix^3$ and ~10% in $alix^1$), branched chains (~40% in $alix^3$ and ~30% in $alix^1$) or polyploidy (~2% in $alix^3$ and ~30% in $alix^1$) (Figs. 3F-H and S7C-S7H). Abscission defects appeared in the majority of both $alix^3$ and $alix^1$ mutant germaria, and never in *wild type* (S3–S4 Tables). Consistently, upon *alix*-RNAi in the germline using *Nanos-GAL4* (*Nanos*-GAL4 or *UAS-Dicer; Nanos-GAL4*) the majority of germaria contained stem cysts in which the fGSC was interconnected to multiple daughter cells via fusome and MRs (S7I-S7K and S5H Figs.). We occasionally detected MBs in stem cysts in $alix^1$ and $alix^3$ mutant germaria (even though MRs predominated), indicating abscission events, and cysts of exactly two cells in the process of pinching off (S7L-S7N Fig.). This is consistent with the model of how 32-cell cysts appear following delayed abscission in fGSCs as previously described [29]. Collectively, these results showed that loss of ALIX caused a delay in abscission in fGSCs with the consequent formation of a high frequency of stem cysts. The fact that cells in stem cysts were interconnected in chains via MRs (Figs. 3F-H, S7C-S7E and S7I-S7J) together with the infrequent observation of fGSC-

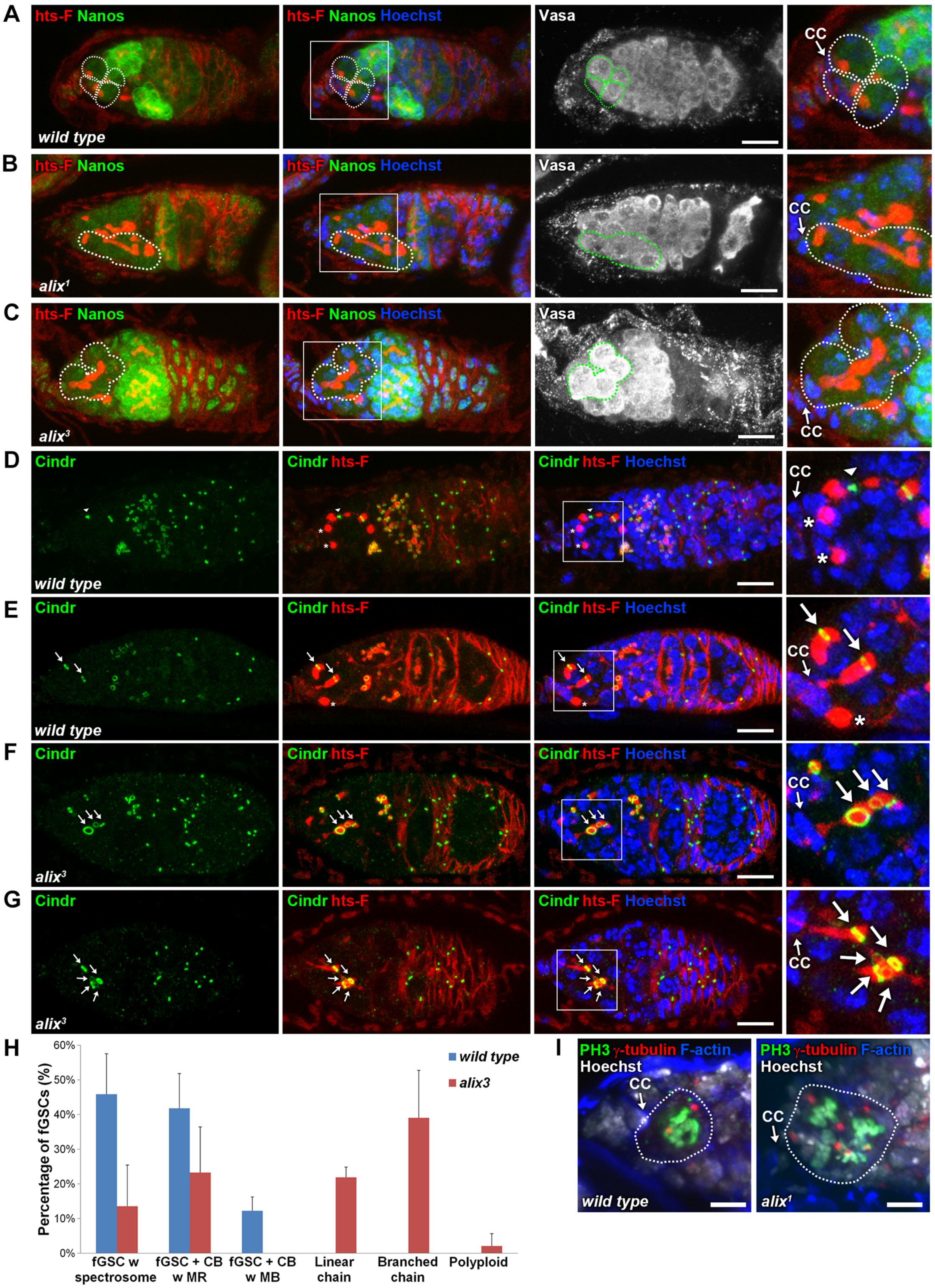


**Figure 3. ALIX controls abscission in *Drosophila* female germline stem cells. (A-C)** Loss of ALIX causes abnormal fGSC division. (A) *Wild type* Nanos-positive fGSCs show normal spectrosome/fusome morphologies (fGSCs and fGSC-CB pairs are outlined). Nanos-positive fGSCs in the anterior tip of *alix*[1] (B) and *alix*[3] (C) mutant germaria are interconnected to chains of daughter cells via abnormally long fusomes (outlined). Ovaries were fixed and stained with antibodies against hts-F (red), Nanos (green) and Vasa (white), and with Hoechst (blue). CC, cap cell. Scale bars represent 10 μm. **(D-G)** Loss of ALIX function causes abscission defects in fGSCs. (D-E) *Wild-type* fGSCs show normal morphologies: single fGSCs with a spectrosome (red, asterisks), fGSC-

CB pairs with an MR (green, arrows) and fusing fusomes (red) and an fGSC-CB pair in abscission with a MB (green, arrowhead) and a fusome with exclamation point morphology (red). (F-G) *alix*$^3$ mutant fGSCs display abnormal morphologies: fGSCs in linear (F) or branched (G) chains via MRs (green, arrows) and fusome (red). Ovaries were fixed and stained with antibodies against Cindr (green) and hts-F (red), and with Hoechst (blue). Scale bars represent 10 μm. **(H)** Graph showing the average percentage of fGSCs with the fGSC phenotypes as described in (D-G) and the Materials and Methods from *wild type* and *alix*$^3$ mutant females. *Wild type*, three independent experiments, n = 61 fGSCs, 22 germaria; *alix*$^3$, three independent experiments, n = 60 fGSCs, 29 germaria. CB, cystoblast; MR, midbody ring; MB, midbody. Data are presented as mean ± STD. See also S3 Table. **(I)** Left: *Wild type* fGSC in mitosis. Right: Four dividing cells in *alix*$^1$ mutant germairum, one of which is an fGSC. Ovaries were fixed and stained with antibodies against phospho-Histone H3 (PH3, green), γ-tubulin (red), with phalloidin to visualize F-actin (blue), and with Hoechst (white). Scale bars represent 5 μm. See also S5–S7 Figs.

doi:10.1371/journal.pgen.1004904.g003

CB pairs with an MB upon loss of ALIX function (Figs. 3H and S7E) suggested that ALIX plays a role in promoting closure of the MR to mediate fGSC abscission. We conclude that ALIX is required for completion of abscission in *Drosophila* fGSCs.

## ALIX controls abscission in *Drosophila* male germline stem cells

We further asked whether ALIX may also be required for abscission in asymmetrically dividing *Drosophila* mGSCs. We stained testes tips from *wild type*, *alix*$^1$ and *alix*$^3$ mutants with antibodies to visualize the hub to which the mGSCs are attached, the fusome, MRs and MBs. In *alix*$^1$ and *alix*$^3$ mutant testes that lack full-length ALIX (S8A Fig.) ~20% of *alix*$^1$ and ~40% of *alix*$^3$ mutant mGSCs were found interconnected to chains of daughter cells by MRs and fusome (S8B-S8E Fig.). These results suggest that ALIX promotes abscission in both female and male GSCs.

## ALIX localizes at midbody rings and midbodies during cytokinesis in *Drosophila* female germline stem cells

To examine the subcellular localization of ALIX during cytokinesis in fGSCs we generated transgenic flies with GFP-tagged ALIX under the control of the *UASp* promoter (*UASp-GFP-ALIX*) and expressed it in fGSCs and germline cysts using *MTD-GAL4* or *Nanos-GAL4*. We then visualized the progressive stages of fGSC cytokinesis using markers for the fusome and MRs/MBs (Cindr) or MTs (α-tubulin). In fGSC-CB pairs in which a small fusome plug had formed within the MR (G1) we detected GFP-ALIX overlapping mainly with the fusome plug (Fig. 4A). At this point we detected anti-parallel MT bundles with a dark zone to which the fusome plug started localizing (S9A Fig.). Then, as the fusome adopted bar morphology in G1/S GFP-ALIX localized at the MR and at this point the MTs were largely degraded (S9B Fig.). GFP-ALIX remained at MRs throughout G1/S, S and early G2 (Figs. 4B and S9B-S9C) and then localized at MBs during abscission (G2) (Fig. 4C). We thus conclude that GFP-ALIX is recruited to the center of the MR and then moves to the MR during G1/S, is detected at MRs throughout cytokinesis progression and then finally localizes to MBs during abscission in *Drosophila* fGSCs. This spatiotemporal dynamics of ALIX during late stages of fGSC cytokinesis is consistent with a role for ALIX in abscission in fGSCs.

## ALIX and Shrub interact and co-localize during cytokinetic abscission in *Drosophila* female germline stem cells

We next asked by which molecular mechanisms ALIX may act during abscission in fGSCs. The ESCRT-III component and CHMP4 orthologue Shrub (*CG8055*) was an interesting candidate to mediate abscission together with ALIX because of the important role of ESCRT-III in promoting membrane scission during cytokinetic abscission and because ALIX directly interacts with and recruits the ESCRT-III subunit CHMP4B to the MB to promote abscission in human cells [13, 15, 52, 53]. The interaction between ALIX and CHMP4B is mediated via a motif within the Bro1 domain of human ALIX (MxxxIxxxL, aa 199–216) and a motif in the CHMP4

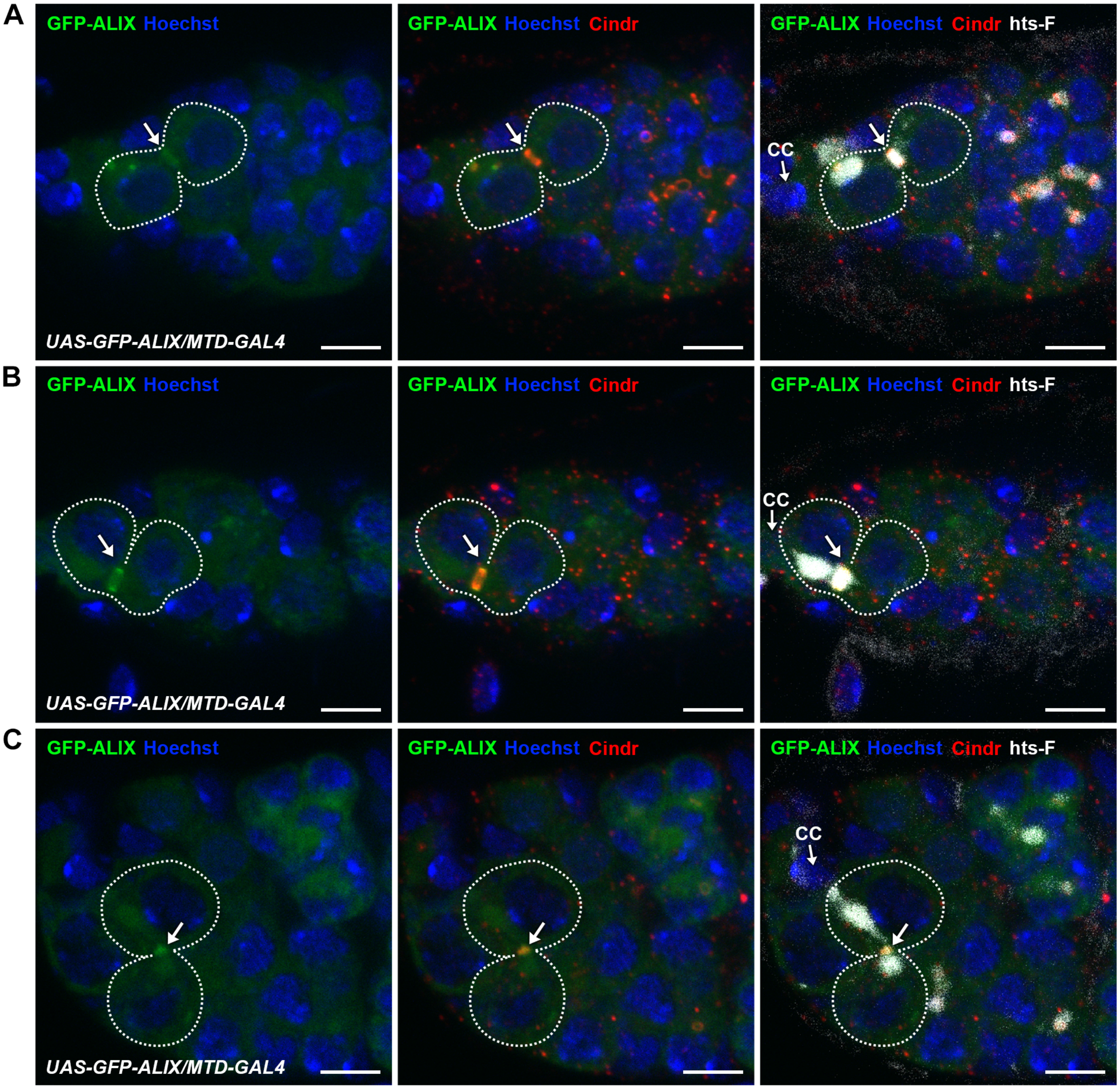


**Figure 4. ALIX localizes at midbody rings and midbodies during cytokinesis in *Drosophila* female germline stem cells. (A-C)** GFP-ALIX localizes at MRs and MBs during fGSC cytokinesis progression. GFP-ALIX was expressed under the control of *MTD-GAL4*. fGSC-CB pairs are outlined and GFP-ALIX is detected at the fusome plug in G1 (A, arrow), at the MR in S phase (B, arrow), and then at the MB during fGSC abscission (C, arrow). Ovaries were fixed and stained with antibodies against Cindr (red) and hts-F (white), and with Hoechst (blue). CC, cap cell. Scale bars represent 5 μm. See also S9 Fig.

doi:10.1371/journal.pgen.1004904.g004

C-terminus (MxxLxxW, aa 214–220) [13, 15, 54]. Importantly, these mutual consensus interaction sites are conserved in *Drosophila* ALIX and Shrub, respectively (ALIX: LxxxIxxxL, aa 198–215 and Shrub: MxxLxxW, aa 218–224) (Fig. 5A) [54]. We therefore tested the possible interaction by co-immunoprecipitation analyses of GFP-tagged Shrub and endogenous ALIX from *Drosophila* Dmel cell lysates. These analyses showed that GFP-Shrub and ALIX indeed were detected in the same complex (Fig. 5B).

We next examined the relative localization of ALIX and Shrub during fGSC cytokinesis. For this purpose GFP-Shrub was expressed using *Nanos-GAL4* and ALIX was detected with our

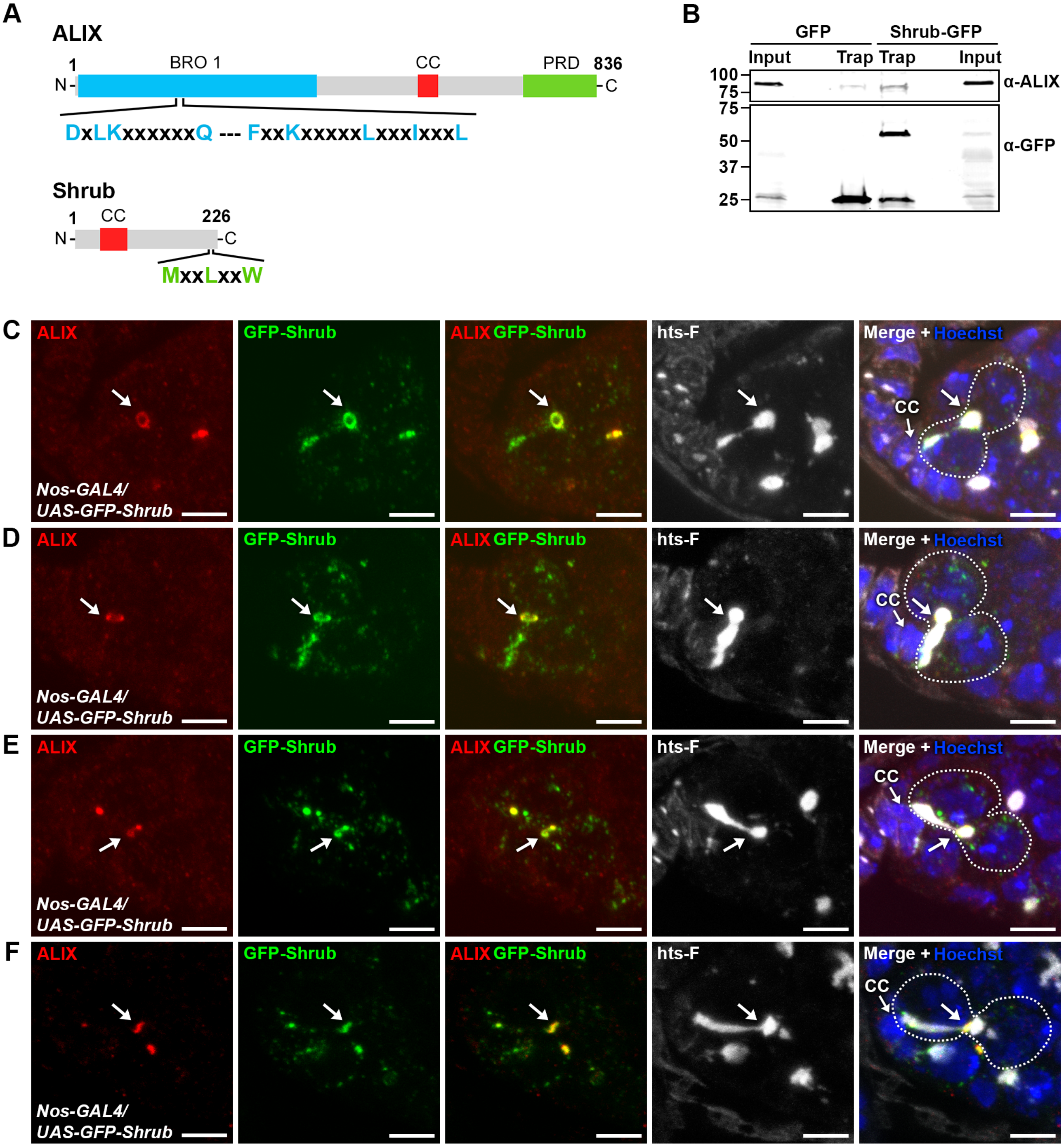


**Figure 5. ALIX co-localizes with Shrub during cytokinesis in *Drosophila* female germline stem cells. (A)** Schematic overview of ALIX and Shrub domain structures and conserved interaction motifs. **(B)** *Drosophila* Dmel cells transiently expressing GFP or Shrub-GFP were subjected to GFP trap immunoprecipiation analysis. ALIX and GFP were detected by immunoblotting. A representative result is shown. **(C-F)** ALIX and GFP-Shrub co-localize at MRs and MBs during fGSC cytokinesis. GFP-Shrub was expressed under the control of *Nanos-GAL4* (*Nos-GAL4*). ALIX co-localizes with GFP-Shrub at MRs in G1/S (C, arrow), S phase (D, arrow) and at MBs during abscission in G2 (E-F, arrows). Ovaries were fixed and stained with antibodies against ALIX (red) and hts-F (white), and with GFP Booster (green) and Hoechst (blue). CC, cap cell. Scale bars represent 5 μm. See also S9 Fig.

doi:10.1371/journal.pgen.1004904.g005

anti-ALIX antibody. Interestingly, GFP-Shrub localized at MRs and MBs during cytokinesis in fGSCs (consistent with observations by [55]) and ALIX co-localized with GFP-Shrub at MRs in G1/S and S phase and then at MBs during abscission in G2 (Fig. 5C-F). Strikingly, GFP-Shrub additionally localized at the fusome (Fig. 5C-D and [55]). Furthermore, ALIX and GFP-Shrub co-localized at bright dot-like structures on the fusome in fGSCs (Fig. 5E). These most likely represented MB remnants that have been reported to be inherited by the fGSC following cytokinesis completion [36]. Consistently, we detected that GFP-ALIX on MB remnants was preferentially retained in fGSCs following abscission (data not shown). We also noted that GFP-Shrub was weakly detected along the membrane at the point that anti-parallel MTs were detected in fGSC-CB pairs in G1 (S9D Fig.) and then accumulated at MRs from G1/S (Figs. 5C-D and S9D). Taken together our results suggested that ALIX and GFP-Shrub co-localize at MRs from G1/S and then at MRs and MBs throughout cytokinetic abscission in *Drosophila* fGSCs.

## ALIX and Shrub coordinately control abscission in *Drosophila* female germline stem cells

We next analyzed the role of Shrub as well as the possible functional relationship between ALIX and Shrub during fGSC cytokinesis. We first performed RNAi-mediated depletion of *shrub* using the *Nanos-GAL4* driver. Control germaria displayed normal fGSC and egg chamber phenotypes (Figs. 6A, 6E, S10A and S10E). Upon *alix*-RNAi about 40% of fGSCs were found in stem cysts (linear or branched) (Fig. 6B and 6E) and ~50% of the egg chambers contained 32 germ cells (S10B and S10E Fig.). Importantly, following *shrub*-RNAi about 45% of fGSCs formed stem cysts (Fig. 6C and 6E), ~10% of the fGSCs were polyploid (Fig. 6E) and ~50% of the egg chambers contained 32 germ cells (Figure S10C and S10E Fig.). Consistently, stem cysts were also present in 70% of heterozygous $shrub^{G5}$/+ mutant germaria (S10F Fig.) suggesting that the stem cysts appeared specifically due to loss of Shrub function. These results showed that loss of Shrub function caused delayed abscission in *Drosophila* fGSCs and that Shrub is required for completion of abscission in these cells.

To test the functional relationship between ALIX and Shrub in fGSCs we performed combined *shrub*- and *alix*-RNAi using *Nanos-GAL4*. We detected about 55% of the fGSCs in stem cysts (Fig. 6D-E), 15% polyploid fGSCs (Fig. 6E) as well as about 40% of egg chambers with 32 germ cells and 15% of egg chambers with more than 32 germ cells (compared to 3–4% in *alix*- or *shrub*-RNAi) (S10D-S10E Fig.). The increased frequency of egg chambers with more than 32 germ cells suggested an even more delayed abscission rate upon combined ALIX and Shrub depletion than following reduction of either ALIX or Shrub levels alone. Consistently, reducing the Shrub levels in the $alix^1$ mutant background ($shrub^{G5}$/+; $alix^1$) gave, in addition to stem cysts, rise to the appearance ~10% of germaria with polyploid fGSCs, ~10% of agametic germaria as well as fewer stem cells per germarium than normal suggesting that reduction of the ALIX and Shrub levels in all cell types in the germarium both caused abscission defects and affected germ cell viability (S10F-S10G Fig.). The fact that reducing the levels of both ALIX and Shrub in fGSCs simultaneously caused even more delayed abscission kinetics in fGSCs as compared to decreasing the levels of either of them alone indicated that ALIX and Shrub are required for the same process to promote abscission in *Drosophila* fGSCs.

## ALIX interacts with Shrub to promote cytokinetic abscission in *Drosophila* female germline stem cells

We next asked whether the complex formation between ALIX and Shrub is important for abscission in fGSCs. In human cells interfering with the interaction between ALIX and CHMP4

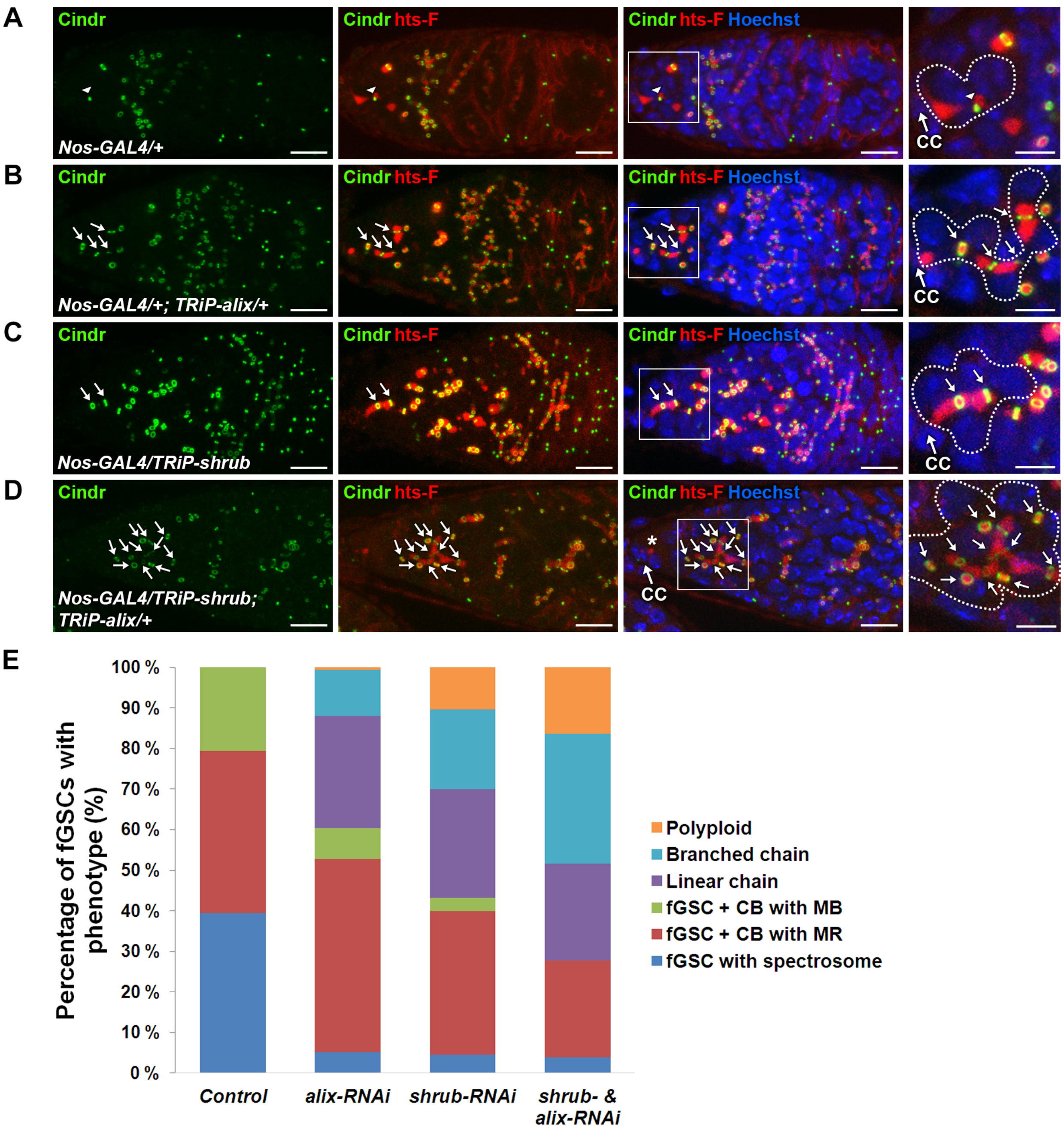


**Figure 6. ALIX and Shrub coordinately control abscission in *Drosophila* female germline stem cells. (A-D)** RNAi-mediated depletion of Shrub and ALIX causes abscission defects in fGSCs. (A) Control fGSC-CB pair in cytokinesis (MB, arrowhead). *alix-RNAi* (B), *shrub-RNAi* (C) and combined *shrub-* and *alix-RNAi* (D) gives rise to fGSCs connected to chains of daughter cells via MRs (green, arrows) and fusome (red). The asterisk in (D) shows a part of the fusome in the fGSC of the stem cyst, a part of which is enlarged. Ovaries were fixed and stained with antibodies against Cindr (green) and hts-F (red) and with Hoechst (blue). CC, cap cell. Scale bars represent 10 μm (full germaria) and 5 μm (enlarged images). **(E)** Graph showing the average percentages of fGSCs with the indicated phenotypes from the genotypes in (A-D). Control, five independent experiments, n = 97 fGSCs, 37 germaria; *alix-RNAi*, five independent experiments, n = 103 fGSCs, 42 germaria; *shrub-RNAi*, four independent experiments, n = 94 fGSCs, 41 germaria; *shrub & alix-RNAi*, three indepdendent experiments, 39 fGSCs, 25 germaria. A systematically significant difference between *control* and either *alix*-RNAi, *shrub*-RNAi or *shrub-RNAi & alix-RNAi* was detected in each experiment ($p<0.005$, Fisher's exact test). CB, cystoblast; MR, midbody ring; MB, midbody. See also S10 Fig.

doi:10.1371/journal.pgen.1004904.g006

causes multi-nucleation and defective midbody morphology [13, 15]. We introduced point mutations in *Drosophila* GFP-ALIX (GFP-ALIX-F198D and GFP-ALIX-I211D) of residues which have previously been shown to mediate the interaction with CHMP4 in human cells [13, 15, 54]. Indeed, we could verify the importance of these residues for the ALIX-Shrub interaction since *wild type* GFP-ALIX co-precipitated substantially more Shrub than the two mutant proteins in GFP trap analyses (Fig. 7A). To further assess the functional importance of the interaction between ALIX and Shrub in abscission in fGSCs we generated flies expressing GFP-ALIX, GFP-ALIX-F198D or GFP-ALIX-I211D using *Nanos-GAL4* either alone or in the *alix*$^{1}$ mutant background. Both GFP-ALIX-F198D and GFP-ALIX-I211D localized at MRs and MBs like *wild type* GFP-ALIX (Figs. 7B and S11A) and their expression per se did not induce the formation of stem cysts (Fig. 7B-C). Importantly, *wild type* GFP-ALIX rescued the fGSC abscission defects in *alix*$^{1}$ mutant germaria from 76% of fGSCs in stem cysts to 22% (Fig. 7C, $p < 0.05$). GFP-ALIX-F198D or GFP-ALIX-I211D could on the other hand not rescue the fGSC abscission defects as 59% and 56% of fGSCs were found in stem cysts following their expression in *alix*$^{1}$ mutant germ cells, respectively (Fig. 7B-C, borderline significant, $p = 0.05$). In agreement, the stem cyst lengths upon expression of GFP-ALIX-F198D or GFP-ALIX-I211D in the *alix*$^{1}$ mutant background were similar to the stem cyst lengths in the *alix*$^{1}$ mutant, whereas they were shorter upon expression of GFP-ALIX (S11B Fig.). These results suggest that ALIX requires the interaction with Shrub to mediate abscission in fGSCs. Moreover, consistent with the stem cyst phenotypes the expression of *wild type* GFP-ALIX in the *alix*$^{1}$ mutant background rescued the number of egg chambers with 32 cells from 49% to 13% ($p < 0.05$), whereas neither GFP-ALIX-F198D nor GFP-ALIX-I211D expression in *alix*$^{1}$ mutant ovaries could rescue the 32-cell phenotype (40% and 39%, respectively, $p < 0.05$) (S11C Fig.). Collectively, these results demonstrate that the direct interaction between ALIX and Shrub is required for completion of abscission with normal kinetics in *Drosophila* fGSCs.

## Discussion

### ALIX and Shrub promote abscission in *Drosophila* female germline stem cells

The mechanisms controlling the kinetics of cytokinetic abscission in different cell types in the context of a multi-cellular organism are not well understood. The *Drosophila* female germline has emerged as a powerful genetically amendable model system to address mechanisms of cytokinetic abscission *in vivo* [29]. In this study we show that the scaffold protein ALIX and the ESCRT-III component Shrub form a complex to mediate completion of cytokinetic abscission in *Drosophila* fGSCs with normal kinetics. Loss of ALIX or/and Shrub function or inhibition of their interaction delays abscission in fGSCs leading to the formation of stem cysts in which the fGSC remains interconnected to chains of daughter cells via MRs. As abscission eventually takes place a cyst of e.g. 2 germ cells may pinch off and subsequently undergo four mitotic divisions to give rise to a germline cyst with 32 germ cells [29]. Consistently, loss of ALIX or/and Shrub or interference with their interaction caused a high frequency of egg chambers with 32 germ cells during *Drosophila* oogenesis. We also found that ALIX controls cytokinetic abscission in both fGSCs and mGSCs and thus that ALIX plays a universal role in cytokinesis during asymmetric GSC division in *Drosophila*. Taken together we thus provide evidence that the ALIX/ESCRT-III pathway is required for normal abscission timing in a living metazoan tissue.

Our results together with findings in other models underline the evolutionary conservation of the ESCRT system and associated proteins in cytokinetic abscission. Specifically, ESCRT-I or ESCRT-III have been implicated in abscission in a subset of *Archaea* (ESCRT-III) [56–58],

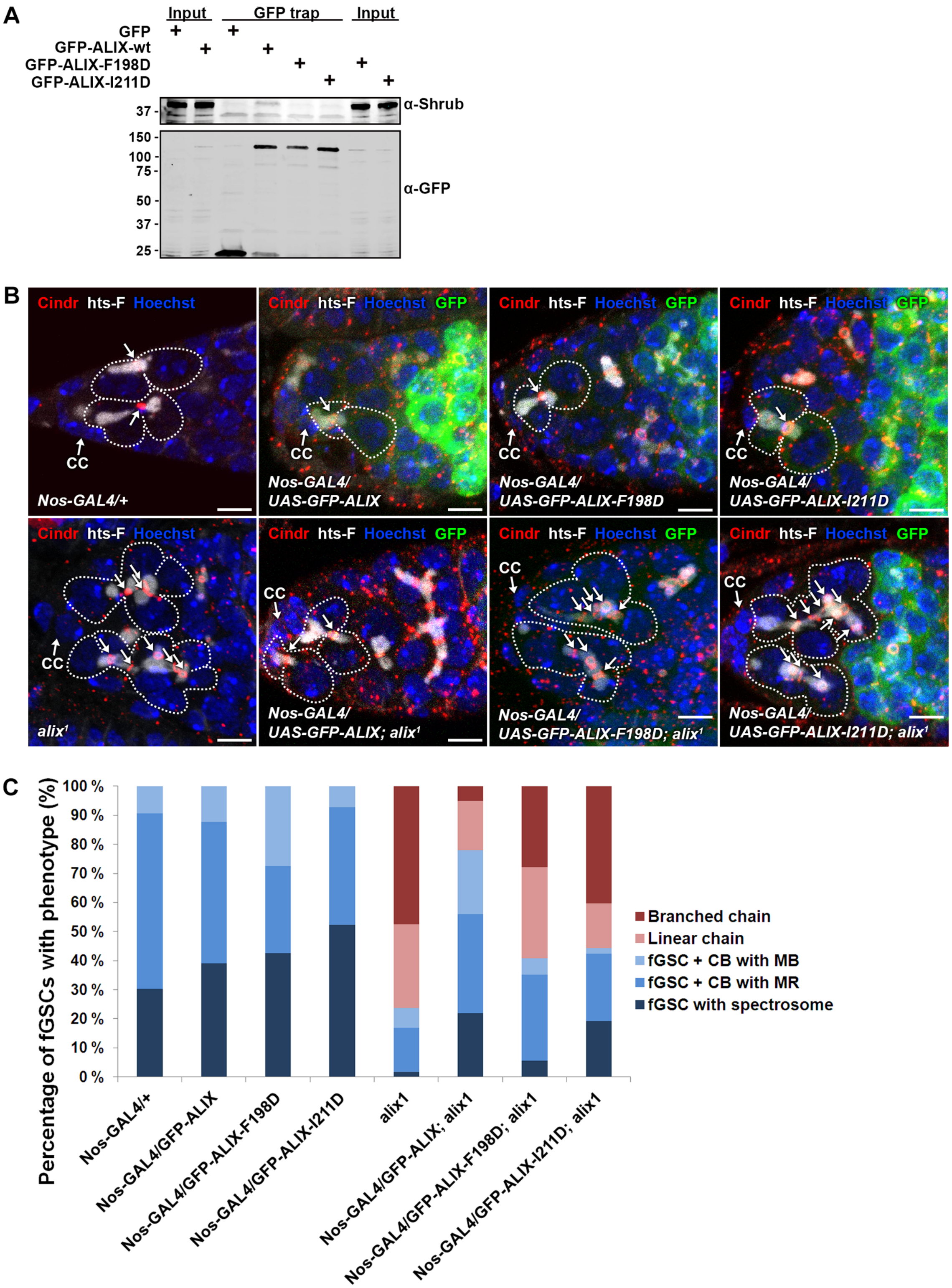


**Figure 7. ALIX interacts with Shrub to promote abscission in *Drosophila* female germline stem cells. (A)** *Drosophila* Dmel cells transiently expressing GFP, *wild type* GFP-ALIX (GFP-ALIX-wt) or the two GFP-ALIX variants containing mutations of amino acids F198 (F198D) or I211 (I211D) were used for GFP trap analysis. Co-immunoprecipitated endogenous Shrub was detected by immunoblotting. Anti-GFP was used to validate the expression levels and levels of precipitated GFP-tagged proteins. A representative result is presented. **(B)** Ovaries of the indicated genotypes were fixed and stained with antibodies against Cindr (red) and hts-F (white) and with Hoechst (blue). fGSC-CB pairs and stem cysts are outlined and MRs indicated with arrows. CC, cap

cell. Scale bars represent 5 μm. **(C)** Graph showing the average percentages of the indicated fGSC phenotypes in germaria of females of the genotypes in (B) from three independent experiments. *Nanos-GAL4/+*, n = 43 fGSCs, 15 germaria; *Nanos-GAL4/UASp-GFP-ALIX*, 41 fGSCs, 15 germaria; *Nanos-GAL4/UASp-GFP-ALIX-F198D*, 40 fGSCs, 15 germaria; *Nanos-GAL4/UASp-GFP-ALIX-I211D*, 42 fGSCs, 15 germaria; *alix*[1], 59 fGSCs, 29 germaria; *Nanos-GAL4/UASp-GFP-ALIX; alix*[1], 59 fGSCs, 28 germaria; *Nanos-GAL4/UASp-GFP-ALIX-F198D*; *alix*[1], 54 fGSCs, 30 germaria; *Nanos-GAL4/UASp-GFP-ALIX-I211D; alix*[1], 52 fGSCs, 29 germaria. See also S11 Fig.

doi:10.1371/journal.pgen.1004904.g007

in *A. thaliana* (elch/tsg101/ESCRT-I) [59] and in *C. elegans* (tsg101/ESCRT-I) [20]. In *S. cerevisiae*, Bro1 (ALIX) and Snf7 (CHMP4/ESCRT-III) have also been suggested to facilitate cytokinesis [60]. In cultured *Drosophila* cells, Shrub/ESCRT-III mediates abscission and in human cells in culture ALIX, TSG101/ESCRT-I and CHMP4B/ESCRT-III promote abscission [9, 11, 13–16]. ALIX and the ESCRT system thus act in an ancient pathway to mediate cytokinetic abscission.

## ALIX in cytokinesis in somatic cells *in vivo*

Despite the fact that we find an essential role of ALIX in promoting cytokinetic abscission during asymmetric GSC division in the *Drosophil* a female and male germlines, we did not detect strong bi-nucleation directly attributed to cytokinesis failure in *Drosophila alix* mutants in the somatic cell types we have examined. This might have multiple explanations. One possibility is that maternally contributed *alix* mRNA may support normal cytokinesis and development. Whereas ALIX and CHMP4B depletion in cultured mammalian cells causes a high frequency of bi- and multi-nucleation [14, 15] it is also possible that cells do not readily become bi-nucleate upon failure of the final step of cytokinetic abscission in the context of a multi-cellular organism. Consistent with our observations of a high frequency of stem cysts upon loss of ALIX and Shrub in the germline, Shrub depletion in cultured *Drosophila* cells resulted in chains of cells interconnected via intercellular bridges/MRs due to multiple rounds of cell division with failed abscission [16]. Moreover, loss of ESCRT-I/tsg101 function in the *C. elegans* embryo did not cause furrow regression [20]. These and our observations suggest that ALIX- and Shrub/ESCRT-depleted cells can halt and are stable at the MR stage for long periods of time and from which cleavage furrows may not easily regress, at least not in these cell types and in the context of a multi-cellular organism. It is also possible that redundant mechanisms contribute to abscission during symmetric cytokinesis in somatic *Drosophila* cells. Further studies should address the general involvement of ALIX and ESCRT-III in cytokinetic abscission in somatic cells *in vivo*.

## Spatiotemporal control of ALIX and Shrub during abscission in *Drosophila* female germline stem cells

Different cell types display different abscission timing, intercellular bridge morphologies and spatiotemporal control of cytokinesis [10, 26, 29]. In fGSCs we found that ALIX and Shrub co-localize throughout late stages of cytokinesis and abscission. In human cells ALIX localizes in the central region of the MB, whereas CHMP4B at first localizes at two cortical ring-like structures adjacent to the central MB region and then progressively distributes also at the constriction zone where it promotes abscission [9–11, 13, 15, 23]. ALIX and CHMP4B are thus found at discrete locations within the intercellular bridge as cells approach abscission in human cultured cells. In contrast, ESCRT-III localizes to a ring-like structure during cytokinesis in *Archaea*, resembling the Shrub localization at MRs we observed in *Drosophila* fGSCs [56, 57]. Moreover, ALIX and Shrub are present at MRs for a much longer time (from G1/S) prior to abscission (in G2) in fGSCs than in human cultured cells. Here, ALIX and CHMP4B are

increasingly recruited about an hour before abscission and then CHMP4B acutely increases at the constriction zones shortly (~30 min) before the abscission event [9, 11].

How may ALIX and Shrub be recruited to the MR/MB in *Drosophila* cells in the absence of CEP55 that is a major recruiter of ALIX and ultimately CHMP4/ESCRT-III in human cells [13, 15]? Curiously, we detect a GPP(3x)Y consensus motif within the *Drosophila* ALIX sequence (**GPP**PGH**Y**, aa 808–814) resembling the CEP55-interacting motif in human ALIX (**GPP**YPT**Y**, aa 800–806). Whether *Drosophila* ALIX is recruited to the MR/MB via a protein (s) interacting with this motif or other domains is presently uncharacterized. Accordingly, alternative pathways of ALIX and ESCRT recruitment have been reported [61–64], as well as suggested in *C. elegans*, where CEP55 is also missing [20]. Further studies are needed to elucidate mechanisms of recruitment and spatiotemporal control of ALIX and ESCRT-III during cytokinesis in fGSCs and different cell types *in vivo*.

## ALIX and Shrub act together to mediate abscission in *Drosophila* female germline stem cells

We found that the direct interaction between ALIX and Shrub is required for completion of abscission with normal kinetics in fGSCs. This is consistent with findings in human cells in which loss of the interaction between ALIX and CHMP4B causes abnormal midbody morphology and multi-nucleation [13, 15]. Following ALIX-mediated recruitment of CHMP4B/ESCRT-III to cortical rings adjacent to the MR in human cells, ESCRT-III extends in spiral-like filaments to promote membrane scission [9–11, 13, 15, 23]. Due to the discrete localizations of ALIX and CHMP4B during abscission in human cells ALIX has been proposed to contribute to ESCRT-III filament nucleation [15, 53]. *In vitro* studies have shown that the interaction between ALIX and CHMP4B may release autoinhibitory intermolecular interactions within both proteins and promote CHMP4B polymerization [54, 65]. Specifically, ALIX dimers can bundle pairs of CHMP4B filaments *in vitro* [65]. Moreover, in yeast, the interaction of the ALIX homologue Bro1 with Snf7 (CHMP4 homologue) enhances the stability of ESCRT-III polymers [66, 67]. There is a high degree of evolutionary conservation of ALIX and ESCRT-III proteins [52–54, 68, 69] and because ALIX and Shrub co-localize and interact to promote abscission in fGSCs it is possible that ALIX can facilitate Shrub filament nucleation and/or polymerization during this process.

Our findings indicate that accurate control of the levels and interaction of ALIX and Shrub ensure proper abscission timing in fGSCs. Their reduced levels or interfering with their complex formation caused delayed abscission kinetics. How cytokinesis is modified to achieve a delay in abscission in *Drosophila* fGSCs and incomplete cytokinesis in germline cysts is not well understood [25–27]. Aurora B plays an important role in controlling abscission timing both in human cells and the *Drosophila* female germline [29, 70, 71]. During *Drosophila* germ cell development Aurora B contributes to mediating a delay of abscission in fGSCs and a block in cytokinesis in germline cysts [29]. Bam expression has also been proposed to block abscission in germline cysts [29, 32, 72, 73]. It will be interesting to investigate mechanisms regulating the levels, activity and complex assembly of ALIX and Shrub and other abscission regulators at MRs/MBs to gain insight into how the abscission machinery is modified to control abscission timing in fGSCs.

We found that intercellular bridge MTs in fGSC-CB pairs were degraded in G1/S when the fusome adopted bar morphology. Abscission in G2 thus appears to occur independently of intercellular bridge MTs in *Drosophila* fGSCs. This has also been described in *C. elegans* embryonic cells where the MR scaffolds the abscission machinery as well as in *Archaea* that lack the

MT cytoskeleton [20, 56, 57]. In mammalian and *Drosophila* S2 cells in culture, on the other hand, intercellular bridge MTs are present until just prior to abscission [9, 11].

## Mechanisms of complete and incomplete cytokinesis in the *Drosophila* female germline

It is interesting to note a resemblance of the stem cysts that appeared upon loss of ALIX and Shrub function to germline cysts in that the MRs remained open for long periods of time similar to RCs. Some modification of ALIX and Shrub levels/recruitment may thus contribute to incomplete cytokinesis in *Drosophila* germline cysts under normal conditions. Because we detected stem cysts in the case when ALIX weakly interacted with Shrub it is also possible that inhibition of their complex assembly/activity may contribute to incomplete cytokinesis in germline cysts. Abscission factors, such as ALIX and Shrub, may thus be modified and/or inhibited during incomplete cytokinesis in germline cysts. Such a scenario has been shown in the mouse male germline where abscission is blocked by inhibition of CEP55-mediated recruitment of the abscission machinery, including ALIX, to stable intercellular bridges [26, 30]. Altogether our data thus suggest that ALIX and Shrub are essential components of the abscission machinery in *Drosophila* GSCs, and we speculate that their absence or inactivation may contribute to incomplete cytokinesis. More insight into molecular mechanisms controlling abscission timing and how the abscission machinery is modified in different cellular contexts will give valuable information about mechanisms controlling complete versus incomplete cytokinesis *in vivo*.

## Conclusions

Summarizing, we here report that a complex between ALIX and Shrub is required for completion of cytokinetic abscission with normal kinetics during asymmetric *Drosophila* GSC division, giving molecular insight into the mechanics of abscission in a developing tissue *in vivo*.

# Materials and Methods

## *Drosophila* stocks and genetics

Fly crosses and experiments were performed at 25°C unless noted otherwise. $w^{1118}$ was used as a *wild type* control. $w^{1118}$, $w^{1118}$; *Nanos-GAL4*, *UAS-Dcr-2*, $w^{1118}$; *Nanos-GAL4*, *y v; attP2*, *TRiP-alix* (*UAS-shRNA-alix*, chr 3, TRiP# HMS00298), *TRiP-shrub* (*UAS-shRNA-shrb*, chr 2, TRiP# HMS01767) [45], *MTD-GAL4* [45], *yw; P{EPgy2}ALiX*$^{EY10362}$, *Df(3R)BSC499/TM6C, Sb*, $w^{1118}$; *Df(3R)BSC739/TM6C*, *Sb* and $w^{*}$*; shrbG5 P{neoFRT}42D/CyO, P{GAL4-twi.G}2.2, P-{UAS-2xEGFP}AH2.2* (*shrub*$^{G5}$/+) were from BDSC (Indiana University) and PBac{WH} ALiXf03094 from Exelixis at Harvard Medical School (referred to as *alix*$^{1}$). The *alix*$^{3}$ allele was generated by imprecise excision of the P-element of the *yw; P{EPgy2}ALiX*$^{EY10362}$ line. The breakpoints were determined by sequencing and this allele lacks 860 bp in the 5' end of the gene (nucleotides 23534881 to 23525741 on 3R missing), thus removing the *alix* gene start codon, exons 1, 2 and most of exon 3. The *FRT82B*, *alix*$^{1}$ and *FRT82B*, *alix*$^{3}$ lines were generated by recombination of to FRT82B chromosomes by standard procedures. The generation of the genomic *alix* rescue lines is described below. The *alix*$^{1}$ and *alix*$^{3}$ alleles were kept as stocks balanced over *TM6B*, *Tb* and *TM6B*, *dfd gfp* chromosomes. *UASp-GFP-Shrub* and *Nanos-GAL4*, *UASp-GFP-Shrub* were generated as described in [55]. *Bam-GAL4* (chr 3) was a kind gift from M. Fuller (Stanford School of Medicine, CA), *hsflp*, *tubulin-GAL4*, *UAS-GFP; FRT82, tubulin-GAL80/TM6B*, *Tb (MARCM82)* was a kind gift from M. Peifer (University of North Carolina).

## Antibodies and reagents

ALIX (*CG12876*) antibodies were generated by immunizing a guinea pig with two peptides (CIQSTYNGASEEEKG-/CERLLDEERDSDNQL-amide) (BioGenes) from the Bro1 domain. Primary antibodies and dilutions for immunofluorescence (IF) or Western blot (WB) were guinea pig anti-ALIX (IF: 1:1000–3000, WB: 1:1000), mouse anti-ALIX (WB: 1:1000, a kind gift from T. Aigaki, Tokyo Metropolitan University, Japan), rabbit anti-Cindr (IF: 1:1000) (Haglund et al., 2010), mouse anti-hts-F (IF: 1:50, 1B1, DSHB), rabbit anti-Shrub (WB: 1:1000, a kind gift from F-G. Bao, University of Massachusetts Medical School, MS [74], mouse anti-α-spectrin (IF: 1:25, 3A9, DSHB), goat anti-Vasa (IF: 1:100, dC-13, Santa Cruz Biotechnology), rabbit anti-Nanos (IF: 1:1000, a kind gift from A. Nakamura, RIKEN Center for Developmental Biology), mouse anti-Bam (IF: 1:10, DSHB), mouse anti-α-tubulin (WB: 1:10,000, Sigma), sheep anti α-tubulin (IF: 1:250, Cytoskeleton), guinea pig anti-Cnn (IF: 1:500, a kind gift from T.C. Kaufman, Indiana University), rabbit anti-phospho-Histone H3 (IF: 1:500, Millipore), rabbit anti-phosphotyrosine (IF: 1:500, Sigma), mouse anti γ-tubulin (IF: 1:500, Sigma), mouse anti-Fasciclin III (FasIII, IF: 1:50, 7G1, DSHB), rabbit anti-phospho-Smad1/5 (Ser463/465) (IF: 1:100, 41D10, Cell Signaling). GFP–Booster_Atto488 (IF: 1:200) was from Chromotek. To visualize F-actin, Alexa Fluor 647 phalloidin (1:50), Alexa Fluor 488 phalloidin (1:100) or rhodamine phalloidin (1:400) (Molecular Probes) were included in secondary antibody incubations. Secondary antibodies were conjugated to Alexa Fluor 488, Alexa Fluor 594 (1:200, Molecular Probes), Cy3 or Cy5 (1:500, Jackson Immunoresearch). DNA was stained using Hoechst 33342 (1μg/μl, Invitrogen). pOT2-ALIX as well as pAGW and pPGW vectors were from the Drosophila Genomics Resource Center (DGRC) (Bloomington, IN). pAc-Shrub-GFP was a kind gift from T. Takeda and D. Glover (University of Cambridge, Cambridge, UK).

## *Drosophila* cell lines

S2 GFP-α-tubulin cells were a kind gift from E. Griffis (University of Dundee, UK) and S2 cells were from ATCC (CRL-1963) (a kind gift from R. Palmer, Umeå University, Sweden). *Drosophila* D.Mel-2 (Dmel) cells (a kind gift from P.P d'Avino and D. Glover, University of Cambridge, Cambridge, UK) were grown in Express Five SFM medium (Gibco) containing 2 mM L-glutamine, 100 U/ml penicillin and 100μg/ml streptomycin and *Drosophila* Schneider 2 (S2) cells were cultured in Schneider's Drosophila Medium (Gibco) supplemented with 10% fetal calf serum, 2 mM L-glutamine, 100 U/ml penicillin and 100μg/ml streptomycin.

## Immunofluorescence staining of *Drosophila* cells and tissues

S2 cells were seeded on coverslips for two hours before 12 min fixation at room temperature in 4% formaldehyde (EM grade, Polysciences) in PHEM buffer (60 mM Pipes pH 6.8, 25 mM Hepes pH 7.0, 10 mM EGTA pH 8.0, 4 mM $MgSO_4$). The cells were then washed three times with PBS and incubated in PBS + 5% BSA + 0,1% Triton X-100) for at least 1 h. Primary antibodies were diluted in PBS + 1% BSA + 0,1% Triton X-100 (PBT) and cells incubated with primary antibodies over night at 4 degrees. Cells were then washed twice in PBT for 15 min and then incubated with secondary antibodies diluted in PBT for 2 hrs at room temperature. They were then washed twice in PBT as before followed by incubation with Hoechst 33342 diluted in PBS to 1μg/μl for 5 min. Cells were finally rinsed with PBS and mounted in Mowiol.

Ovaries or testes were dissected in PBS and fixed using 4% formaldehyde (EM grade, Polysciences) for 30 min either on ice (all samples including anti-Cindr antibodies) or at room temperature (RT) (prior to anti-α-tubulin staining). Tissues were subjected to permeabilization (3 × 15min) and blocking (30 min) in PBS + 0.3% bovine serum albumin (BSA) + 0.3% Triton X- 100 (PBT) at RT and then incubated with primary antibodies diluted in PBT at 4°C over

night. Samples were then washed three times 15 min in PBT, incubated with secondary antibodies diluted in PBT for 2 hrs at room temperature followed by three 15 min washes in PBT. For DNA staining, samples were subsequently stained with Hoechst 33342 (1 μg/μl) diluted in PBS for 10 min. Samples were mounted in anti-fading mounting medium (Prolong Antifade, Molecular Probes or Vectashield, Vector laboratories). For anti-ALIX staining, ovaries were fixed in ice-cold methanol for 7 min and subsequently stained as above with the addition of GFP-Booster (1:200) in the secondary antibody solution. For p-Mad detection the ovaries were fixed for 40 min in 4% formaldehyde with phosphatase inhibitor cocktail (Sigma, 1:200) and stained according to the protocol by Luo *et. al.* [75].

## Confocal microscopy

Images were captured using Zeiss LSM 780, Zeiss LSM 710 or Zeiss LSM 5 DUO laser scanning confocal microscopes (Carl Zeiss, Inc.) equipped with NeoFluar 63×/1.4 NA and 100×/1.45 NA oil immersion and Plan Apochromat 20×/0.8 NA objectives at 20°C. Image processing and analysis were done using the Zeiss LSM 510 (Version 3.2, Carl Zeiss, Inc.) and Zen 2009 softwares and Adobe Photoshop CS4 (Adobe). Images are planar projections of sections from z-stacks of germaria unless otherwise noted.

## Quantification of female germ stem cell and egg chamber phenotypes

Ovaries of 2 to 4-day-old females (unless otherwise noted) that had been fed with yeast paste and kept with a couple of males for 2 days were dissected, fixed and stained with antibodies to visualize the fusome (hts-F), MRs/MBs (Cindr), RCs (pTyr)/F-actin (fluorescently labeled phalloidin) and nuclei (Hoechst) as described above. Confocal z-stacks of germaria were acquired at the confocal microscope and fGSC phenotypes were analyzed from z-stacks and three-dimensional reconstructions of z-stacks. fGSC identity was determined based on its anterior localization in the germarium, its fusome morphology and contact with the cap cells. Phenotype scoring was based on the fusome morphology, presence, absence, number and position of Cindr-positive MRs/MBs, cell-cell boundaries and nuclei. We categorized fGSCs into normal morphologies: (i) fGSCs with a spherical spectrosome, (ii) fGSC-CB pairs with an MR (includes plug, bar, dumbbell and fusing fusome morphologies) and (iii) fGSC-CB pairs in abscission with an MB between them (exclamation point fusome) as well as abnormal abscission-defective morphologies: (iv) linear chains of cells interconnected via fusome and MRs, (v) branched chains or (vi) polyploid, bi- or multinucleate fGSCs. Egg chamber phenotypes were scored at the microscope based on the number of RCs to the oocyte and the number of germ cell nuclei.

## Statistical analyses

To examine whether differences between controls and *alix*$^1$ or *alix*$^3$ germaria were significant within experiments, each germarium was classified as either normal or non-normal (the latter being the case if at least one non-normal phenotype was present—linear, branched or polyploid). Fisher's exact test was then used to determine significance. To test whether differences of fGSC phenotypes (classified as above) or egg chamber phenotypes between *Nos-GAL4/GFP-ALIX* and *alix*$^1$, *Nos-GAL4/GFP-ALIX-F198D; alix*$^1$ or *Nos-GAL4/GFP-ALIX-I211D; alix*$^1$ ovaries were significant we used a mixed factor model with each experiment as random factor.

## Constructs

To generate N-terminally GFP-tagged *alix*, a PCR fragment corresponding to the whole-length *alix* cDNA (except the START codon) was amplified from a cDNA clone from the BDGP Gold

cDNA Collection (DGRC, Bloomington, IN) using the primers 5'AATGGATCCGGTCGAAG-TTTCTGGGCGTGCCG3' and 5'AATGCGGCCGCTTACCAGCCAGGTGGCTTCTG3' and the Phusion High-Fidelity PCR Kit (New England Biolabs). The *alix* cDNA was purified using the QIAquick PCR Purification Kit (Qiagen), and cloned into the pENTR1A Gateway entry vector using the T4 DNA ligase (Roche). The *alix* gene was then transferred by LR recombination using the Gateway LR clonase II enzyme mix (Invitrogen) to the pPGW (for generation of fly lines) or pAGW (for cell lines) destination vectors (DGRC, Bloomington, IN). Site-directed *in vitro* mutagenesis was used to introduce point mutations in the pENTR1A-*alix* vector using primers containing the specific mutations and the Phusion High-Fidelity PCR Kit (New England Biolabs). For generating ALIX-F198D the primers 5'CCAAGCGCAGGAGGTTGA-CATTCTGAAGGCAATTAAGG3' and 5'CCTTAATTGCCTTCAGAATGTCAACCTCCTG-CGCTTGG3' were used, and for ALIX-I211D the primers 5'CTTGAAGGACCAGGA-CATCGCCAAGCTTTGCTGC3' and 5'GCAGCAAAGCTTGGCGATGTCCTGGTCCTT-CAAG3' were used. The plasmid was then treated with DpnI (New England Biolabs) for one hour at 37°C after PCR amplification. The mutated *alix* cDNAs were then transferred to the pPGW (for fly lines) and pAGW (for cell lines) destination vectors by LR recombination

## Generation of transgenic *Drosophila* lines

The transgenic *UASp-GFP-ALIX*, *UASp-GFP-ALIX-F198D* and *UASp-GFP-ALIX-I211D Drosophila* lines were generated by P-element transformation performed by BestGene Inc. The expression of GFP-ALIX was verified by Western blot analysis.

## Co-immunoprecipitation

Approximately 1 hour before transfection, $8 \times 10^6$ Dmel cells were seeded in 10 cm plates. The cells were transiently transfected for 48 hours with 2,5 μg pAGW (empty GFP) or 5 μg pAc-Shrub-GFP, pAGW-ALIX-wt, pAGW-ALIX-F198D or pAGW-ALIX-I211D using FuGene HD according to the manufacturer's instructions (Promega). Enrichment of mitotic cells was obtained by MG132 treatment (25 μM, 5 hours) as previously described [76]. Cells were used for GFP trap immunoprecipitation analysis performed in line with the protocol provided by the supplier (ChromoTek). The cells were lysed in 200 μl Lysis buffer (10 mM Tris-HCl pH 7.5, 150 mM NaCl, 0.5 mM EDTA, 0.5% NP-40) supplemented with 1:50 protease inhibitor cocktail (Roche), 1:50 phosphatase inhibitor cocktail 2 (Sigma-Aldrich) and 2 mM N-ethylmalemide (Sigma-Aldrich) on ice for 30 minutes with extensive mixing every 10 minutes. Nuclei and cell debris were cleared by centrifugation (20,000*g*, 10 minutes, 4°C), before the lysate was diluted to 1000 μl with Washing buffer (10 mM Tris-HCl pH = 7.5, 150 mM NaCl, 0.5 mM EDTA) and incubated with pre-washed GFP trap beads (30 μl) for 1 hour at 4°C. The beads and associated proteins were washed three times using Washing buffer and next boiled in SDS sample buffer containing 100 mM DTT for 10 minutes to elute associated proteins. The eluted proteins were subjected to SDS-PAGE, followed by Western blot to detect ALIX, Shrub or GFP.

## Western blot analyses

*Drosophila* tissues were collected and homogenized in ice-cold lysis buffer (50 mM Tris pH 8, 150 mM NaCl, 0.5% NP-40 or 50 mM Hepes, 150 mM NaCl, 1 mM EDTA, 1 mM EGTA, 10% glycerol, 1% Triton X-100, 25 mM NaF, 10μM $ZnCl_2$) containing protease inhibitor cocktail (Complete, EDTA-free, Roche). Lysates were cleared by centrifugation for 15 min at 13,000 rpm and 4°C. Equal amounts of protein were mixed with Laemmli buffer containing 50 mM DTT, denatured by boiling and subjected to SDS-PAGE and transferred to either

nitrocellulose or PVDF membranes. Nitrocellulose membranes were blocked in PBS/5% milk at 4°C over night followed by incubation with primary antibodies diluted in PBS/5% BSA for 1h 30 min or over night. Membranes were then washed three times in PBS/0.01% Tween-20, followed by incubation 1 h with secondary HRP-conjugated anti-rabbit and anti-mouse antibodies (1:5000) (Jackson ImmunoResearch). Following three further washes in PBS/0.01% Tween-20 and one wash in PBS, chemiluminescent (WestPico, PIERCE) signal was detected on film (Amersham Hyperfilm). PVDF membranes were blocked (by drying), re-wet in PBS/0.01% Tween-20, incubated with primary antibodies overnight at 4°C, rinsed three times in PBS/0.01% Tween-20, incubated with fluorescently labelled secondary antibodies (LI-COR Biosciences GmbH) and washed twice in PBS/0.01% Tween-20 and once in PBS followed by scanning using the Odyssey Developer (LI-COR Biosciences GmbH).

## RNAi-mediated depletion in *Drosophila* female germ cells

For RNAi-mediated gene silencing in germ cells, *MTD-GAL4*, *Nanos-GAL4*, *UAS-Dicer; Nanos-Gal4* or *Bam-GAL4* drivers were crossed to control (*yv; attP2*), *TRiP-alix-RNAi*, *TRiP-shrub-RNAi* or *TRiP-shrub-RNAi; TRiP-alix-RNAi* flies as described. For all *RNAi* experiments, young female offspring were fed with yeast paste and kept with a couple of males for 2 days at 25°C. Ovaries of 2–4-day-old females were dissected, fixed, and stained as described above.

## Quantification of *Drosophila* male germ stem cell phenotypes

0–3 day old males were dissected and stained with antibodies as described above to label midbody rings and midbodies (Cindr), the fusome (α-spectrin), hub (Fasciclin III) and germ cells (Vasa). Confocal z-stacks of testes tips were acquired at the confocal microscope and mGSC phenotypes were analyzed from z-stacks and three-dimensional reconstructions of z-stacks. mGSC identity was determined based on proximity to the hub. Phenotype scoring was based on the fusome morphology, presence, absence, number and position of Cindr-positive MRs/MBs, Vasa staining and nuclei.

## Clonal analyses in follicle cell epithelia

For clonal analysis in the follicle cell epithelium, *MARCM82* females were crossed to *FRT82* and *FRT82*, *alix*$^{3}$*/TM6B, Tb* and *FRT82*, *alix*$^{1}$*/TM6B, Tb* males. L3 larvae were subjected to two heat-shocks at 37°C for 1 h. Newly hatched females were fed with yeast paste for 2 days in the presence of a couple of males. Ovaries were then dissected and stained to visualize F-actin and nuclei (Hoechst) as described above.

## Generation of genomic rescue lines, rescue analyses and complementation tests

Genomic rescue constructs (BAC CH322–119C06, comprising 20339 bp from *23513227 to 23533565 of chromosome arm 3R (short-alix-rescue*, *alix-s), and* BAC CH321–50C24 comprising 85562 bp from *23500943 to 23586504 of chromosome arm 3R (long-alix-rescue*, *alix-l*) in the vector attB-P[acman]-Cm$^{R}$-BW (http://bacpac.chori.org/home.htm) were injected into strains y1 *w*$^{1118}$*; PBac{y+-attP-9A}VK00018* (BDSC# 9736, insertion site 53B2) and y1 *w*$^{1118}$*; PBac{y +-attP-3B}VK00037* (BDSC# 9752, insertion site 22A3) and integrated into predetermined attP docking sites in the genome using PhiC31 integrase-mediated germline transformation. The methodology is decribed in "Versatile P[acman] BAC libraries for transgenesis studies in *Drosophila melanogaster*" [77]. The injection of the constructs into *Drosophila* embryos was performed by BestGene (http://www.thebestgene.com/). Males with integrated constructs were

obtained from BestGene, balanced and crossed to generate *CH322–119C06/CyO; alix$^1$/TM6B, Tb* (*alix-s/CyO; alix$^1$/TM6B, Tb*), *CH322–119C06/CyO; alix$^3$/TM6B, Tb* (*alix-s/CyO; alix$^3$/TM6B, Tb*), *CH321–50C24/CyO; alix$^1$/TM6B, Tb* (*alix-l/CyO; alix$^1$/TM6B, Tb*) and *CH321–50C24/CyO; alix$^3$/TM6B, Tb* (*alix-l/CyO; alix$^3$/TM6B, Tb*) stocks.

For complementation tests the *alix$^1$* and *alix$^3$* alleles were crossed to the deficiencies and to each other. In both rescue analyses and complementation tests, young females of the indicated genotypes were collected, fed with yeast paste and kept with a couple of males for 2 days. Ovaries of 2–4 day-old flies were dissected, fixed and stained to visualize F-actin and nuclei and egg chamber phenotypes were quantified as described above.

## Egg lay and hatch rate assays

Flies used for fertility tests were 4–7 days old and kept separately with yeast paste for a couple of days before being crossed. *Wild type* or *alix$^1$* mutant virgin females were crossed to *wild type* or *alix$^1$* mutant males as indicated. Eggs were collected on apple juice agar plates for 18 hours three times for each cross in three independent experiments. The eggs were counted after each egg lay to determine the egg lay rate. Hatch rates were determined by quantifying the hatched versus unhatched eggs under a dissecting microscope after eggs had developed for 24–30 hours. The experiments were conducted at 25°C.

## *Drosophila* embryo stainings

Embryo collection, permeabilization and fixation were based on the protocol described by Rothwell and Sullivan [78]. The *Drosophila melanogaster* flies were put on apple juice agar with yeast for egg lay at 25°C overnight. The embryos were dislodged from the agar into a nylon mesh/falcon basket using PBS + 0.02% Triton X-100, and dechorionated by shaking them in a 50% commercial bleach solution until agglutination of the embryos (1–3 min). The dechorionated embryos were extensively rinsed with PBS + 0.02% Triton X-100, and blotted dry on paper towels. The embryos were transferred from the nylon mesh and to a small flask with 5 mL heptane. An equal amount of 4% formaldehyde in PBS was added, and the two-phase mixture was incubated with vigorous shaking for 17 minutes. The embryos were now between the two phases. The formaldehyde phase was removed and replaced with methanol, and the embryos were gently shaken for 1 minute with gentle heating for removal of the vitelline membrane. The heptane phase was removed along with the embryos still remaining in the interphase. The embryos that sank to the bottom of the flask were washed three times in methanol and stored at -20°C. Immunofluorescent staining of embryos was performed as follows. The embryos were rehydrated by first putting them in 3:4 methanol and 1:4 4% formaldehyde in PBS for 2 minutes, and then 1:4 methanol and 3:4 formaldehyde for 5 minutes. Post fixation was done for 10 minutes in 4% formaldehyde, before the embryos were rinsed six times using PBS with 1% BSA and 0.05% Triton X-100. The embryos were incubated with α-spectrin antibodies (1:25, DSHB) over night at 4°C. After incubation the embryos were rinsed three times and washed for one hour with PBS with 1% BSA and 0.05% Triton X-100, and then incubated with secondary antibody for two hours. The antibodies were diluted in PBS with 1% BSA and 0.05% Triton X-100. The embryos were again rinsed three times and washed for one hour with PBS with 1% BSA and 0.05% Triton X-100, before being labeled with Hoechst 33342 (2 μL/mL) for 10 minutes, and then rinsed 3 times in PBS to remove detergent. The embryos were mounted using Vectashield (Vector laboratories). For quantifications of mono- and binucleate cells, images of homozygous *wild type*, *alix$^1$* and *alix$^3$* mutant stage 16 embryos were captured at the confocal microscope and more than 1000 cells analyzed for each genotype.

## Supporting Information

**S1 Fig. *Drosophila* ALIX protein expression and spatiotemporal dynamics during division of cultured *Drosophila* cells. (A-C)** ALIX co-localizes with Centrosomin (Cnn) at centrosomes in meta-, anaphase and early telophase (A', B' and C'). No signal is detected at centrosomes using the pre-immune (pre-im) serum (A, B and C). **(D)** ALIX co-localizes with Cnn at centrosomes and in addition appears at the intercellular bridge in mid telophase where it overlaps with the mitotic spindle (D'). No signal is detected at centrosomes nor at the intercellular bridge using the pre-immune serum (D). **(E)** In late telophase/cytokinesis, ALIX localizes to the dark region in the α-tubulin staining at the centre of the intercellular bridge, indicating its localization at the midbody ring (E'). ALIX also shows a vesicular pattern within the cell at this stage (E'). No signal is detected at the midbody ring using the pre-immune serum (E). A weak vesicular pattern detected, but is much weaker than in (E'). In all panels, S2 cells were fixed and stained with antibodies against ALIX (red), Cnn (green) and α-tubulin (white), and with Hoechst (blue). Images in all panels were captured with the same intensity. Scale bars represent 5 μm.
(TIF)

**S2 Fig. ALIX expression in *Drosophila* tissues and phenotypes following loss of ALIX function in somatic *Drosophila* cell types. (A)** Western blot showing ALIX expression levels in *Drosophila* embryos, L3 larvae, pupae, adult males and females as well as testes and ovaries. α-tubulin was used as a loading control. **(B)** Western blot showing loss of ALIX protein in $alix^1$ mutant males and females. Heterozygote $alix^1$*/TM6B, Tb* males and females show reduced protein levels compared to *wild type*. α-tubulin was used as a loading control. **(C)** Left: Image of *wild type* stage 16 embryonic epithelium. Middle and right: Images of homozygous $alix^1$ and $alix^3$ mutant stage 16 embryonic epithelia. Embryos were fixed and stained with antibodies against α-spectrin (red) and with Hoechst (green). More than 1000 cells from five embryos of each genotype were analyzed for the presence of mono- and bi-nucelate cells and no evident bi-nucleation could be detected for any of the genotypes. Scale bars represent 10 μm. **(D)** Left: Image of *wild type* follicle cell epithelium of stage 6 egg chamber. Middle and right: Images of $alix^1$ and $alix^3$ mutant follicle cell epithelia of stage 6 egg chambers. Bi-nucleate cells are indicated with asterisks. Ovaries were fixed and stained to visualize F-actin (red) and nuclei (white, Hoechst). Scale bars represent 5 μm. See also S1 Table. **(E)** Left: Stage 10 EC with GFP-positive mono-nucleate control follicle cell clones. Middle and right: $alix^3$ and $alix^1$ mutant GFP-positive clones with bi-nucleate cells (asterisks). Ovaries were fixed and stained to visualize F-actin (red) and nuclei (Hoechst, blue). Scale bars represent 20 μm. See also S2 Table. **(F)** Left: Stage 14 EC with GFP-positive mononucleate control follicle cell clones. Middle and right: $alix^3$ and $alix^1$ mutant GFP-positive clones with bi-nucleate cells (asterisks). The bi-nucleation in the *alix* mutant clones may arise via alternative mechanisms. It is possible that loss of ALIX function leads to loss of the connection of the stable intercellular bridge between follicle cells with the plasma membrane as the egg chamber develops from stage 10 to stage 14 and thus that ALIX is required to maintain separate cells at late stages of oogenesis in somatic *Drosophila* follicle cells. Alternatively, the bi-nucleation could be caused by abscission failure, but an abscission event in follicle cells at late stages of oogenesis has not, to our knowledge, been described as the stable intercellular bridges formed via incomplete cytokinesis in the follicle epithelium are thought to persist throughout *Drosophila* oogenesis [26, 79, 80]. Ovaries were fixed and stained to visualize F-actin (red) and nuclei (Hoechst, blue). Scale bars represent 20 μm. See also S2 Table.
(TIF)

**S3 Fig. Loss of ALIX causes severely reduced female fertility and defects in oogenesis in *Drosophila melanogaster*. (A)** Graph showing the average egg lay rates for *wild type* and $alix^1$

mutant females crossed to either *wild type* or *alix*$^1$ mutant males from three independent experiments. Data are presented as mean ± STD. **(B)** Graph showing average hatch rates for the eggs laid in the crosses from the three independent experiments in (B). Data are presented as mean ± STD. **(C)** Western blot showing expression of ALIX protein in *wild type* ovaries and loss of ALIX protein in ovaries of *alix*$^1$ homozygote mutant females, of females in which the *alix*$^1$ allele is combined with two different deficiences (*alix*$^1$*/Df(3R)BSC499, alix*$^1$*/Df499* and *alix*$^1$*/Df(3R)BSC739, alix*$^1$*/Df739*) or with the *alix*$^3$ allele (*alix*$^1$*/alix*$^3$), of *alix*$^3$ homozygote mutant females, or females in which the *alix*$^3$ allele is combined with the two different deficiences (*alix*$^3$*/Df499* and *alix*$^3$*/Df739*). α-tubulin served as a control for protein loading. **(D)** Images showing four ring canals (arrows) to the oocyte in a *wild type* egg chamber and five ring canals (arrows) to the oocyte in egg chambers of the genotypes in (C). Ovaries were fixed and stained to visualize F-actin (white). Scale bars represent 5 μm. **(E)** Graph showing the percentages of egg chambers with 16, 32 or more germ cells the indicated genotypes in (C-D). *Wild type*, three independent experiments, n = 362 egg chambers; *alix*$^1$, two independent experiments, n = 154 egg chambers; *alix*$^1$*/Df(3R)BSC499*, two independent experiments, n = 242 egg chambers; *alix*$^1$*/Df(3R)BSC739*, two independent experiments, n = 38; *alix*$^1$*/alix*$^3$, one experiment, n = 42 egg chambers, *alix*$^3$, two independent experiments, n = 150 egg chambers, *alix*$^3$*/Df(3R) BSC499*, two independent experiments, n = 139; *alix*$^3$*/Df(3R)BSC739*, one experiment, n = 88 egg chambers. Data are presented as mean ± STD.
(TIF)

**S4 Fig. Rescue of the *alix*$^1$ and *alix*$^3$ mutant egg chamber phenotypes and appearance of egg chambers with increased germ cell number upon germline-specific *alix* depletion. (A)** Western blot showing ALIX expression in *wild type* ovaries, lack of ALIX protein in *alix*$^1$ homozygote mutant ovaries and ALIX expression in ovaries of two lines with one copy of either of the two genomic rescue constructs (*short-alix-rescue*, *alix-s; long alix-rescue*, *alix-l*) in the *alix*$^1$ mutant background (*alix-s/CyO; alix*$^1$ and *alix-l/CyO; alix*$^1$). Levels of α-tubulin show equal protein loading. Rescue constructs and lines are described in the Materials and Methods. **(B)** Images showing four ring canals (arrows) to the oocyte in a *wild type* egg chamber, five ring canals to the oocyte (arrows) in an *alix*$^1$ mutant egg chamber and four ring canals (arrows) to the oocyte upon reexpression of ALIX in the *alix*$^1$ mutant background from either of the two rescue constructs (*alix-s/CyO; alix*$^1$ and *alix-l/CyO; alix*$^1$). Ovaries were fixed and stained to visualize F-actin (white). Images are planar projections of several sections of a z-stack. Scale bars represent 5 μm. **(C)** Graph showing the average percentage of egg chambers with 16, 32 or more germ cells of the genotypes in (A) and (B). *Wild type*, three independent experiments, n = 326 egg chambers; *alix*$^1$, three independent experiments, n = 236 egg chambers; *alix-s/CyO; alix*$^1$, three independent experiments, n = 301 egg chambers; *alix-l/CyO; alix*$^1$, three independent experiments, n = 226 egg chambers. Data are presented as mean ± STD. **(D)** Western blot showing ALIX expression in *wild type* ovaries, lack of ALIX protein in *alix*$^3$ homozygote mutant ovaries and ALIX expression in ovaries from two lines with one copy of either of the two genomic rescue constructs described in (A) in the *alix*$^3$ mutant background (*alix-s/CyO; alix*$^3$ and *alix-l/CyO; alix*$^3$). Levels of α-tubulin show equal protein loading. **(E)** Images showing four ring canals to the oocyte (arrows) in a *wild type* egg chamber, five ring canals (arrows) to the oocyte in an *alix*$^3$ mutant egg chamber and four ring canals (arrows) to the oocyte upon reexpression of ALIX in the *alix*$^3$ mutant background from either of the two rescue constructs (*alix-s/CyO; alix*$^3$ and *alix-l/CyO; alix*$^3$). Ovaries were fixed and stained to visualize F-actin (white). Scale bars represent 5 μm. **(F)** Graph showing the average percentage of egg chambers with 16, 32 or more germ cells from the genotypes in (D) and (E). *Wild type*, three independent experiments, n = 340 egg chambers; *alix*$^3$, three independent experiments,

n = 275 egg chambers; *alix-s/CyO; alix*[3], three independent experiments, n = 310 egg chambers; *alix-l/CyO; alix*[3], two independent experiments, n = 235 egg chambers. Data are presented as mean ± STD. **(G)** Western blot showing ALIX expression in control ovaries (*MTD-GAL4/+*) and reduced levels of ALIX protein in ovaries in which RNAi-mediated gene silencing was performed in germ cells using *MTD-GAL4* and the *TRiP-alix*-RNAi line (*alix-RNAi*). **(H)** Images showing four ring canals (arrows) to the oocyte of a control egg chamber and five ring canals (arrows) to the oocyte in an *alix-RNAi* egg chamber. Ovaries were fixed and stained to visualize F-actin (white). Images are planar projections of several sections from z-stacks. Scale bars represent 10 μm. **(I)** Graph showing the average percentage of egg chambers with 16, 32 or other phenotypes from *control* and *alix-RNAi* females. *Control*, four experiments, n = 423 egg chambers; *alix-RNAi*, four experiments, n = 602 egg chambers. Data are presented as mean ± STD. (TIF)

**S5 Fig. Loss of ALIX in *Drosophila* female germline stem cells gives rise to egg chambers with 32 germ cells and abscission defects in these cells. (A)** *Control* (*Nos-GAL4/+*) egg chamber with four ring canals (arrows) to the oocyte (left image) and egg chamber with *alix-RNAi* (*Nos-GAL4/+; TRiP-alix/+*) expression in fGSCs (right image) with five ring canals (arrows) to the oocyte are shown. *Nos*, *Nanos*; *TRiP-alix* = *alix*-RNAi line from TRiP. **(B)** *Control* (*Dcr2/+; Nos-GAL4/+*) egg chamber with four ring canals (arrows) to the oocyte (left image) and egg chamber with *alix-RNAi* and Dicer (*Dcr2/+; Nos-GAL4/+; TRiP-alix/+*) expression (right image) with five ring canals to the oocyte are shown. *Dcr2*, *Dicer 2*. **(C)** Egg chambers from females with the genotypes *Bam-GAL4/+* (left image) and *Bam-GAL4/TRiP-alix* (right image) with four ring canals (arrows) to the oocyte are shown. In (A-C) ovaries were fixed and stained with fluorescently labeled phalloidin (green) and with Hoechst (not shown). Scale bars represent 20 μm. **(D)** Graph showing the average percentage of egg chambers with 16 or 32 or more germ cells for the genotypes in (A-C). *Nos-Gal4/+*, three independent experiments, n = 181 egg chambers; *Nos-Gal4/+; TRiP-alix/+*, three independent experiments, n = 159 egg chambers; *Dcr2/+; Nos-GAL4/+*, three independent experiments, n = 149 egg chambers; *Dcr2/+; Nos-GAL4/+; TRiP-alix /+*, three independent experiments, n = 146; *Bam-GAL4/+*, three independent experiments, n = 179 egg chambers; *Bam-GAL4/TRiP-alix*, three independent experiments, n = 192 egg chambers. Data are presented as mean ± STD. **(E)** Germarium from *control* female (*Nos-GAL4/+*) with Nanos-positive fGSCs with spectrosomes (left image) and germarium with *alix-RNAi* expression in germ cells, including fGSCs, using *Nos-GAL4* (*Nos-GAL4/+; TRiP-alix/+*) (right image) are shown. In the right image an fGSC interconnected to three other Nanos-positive cells via fusome is seen in the anterior tip of the germarium. **(F)** Germarium from *control* female (*Dcr2/+; Nos-GAL4/+*) (left image) with Nanos-positive fGSCs with spectrosomes and germarium in which *alix-RNAi* and Dicer were expressed (*Dcr2/+; Nos-GAL4/+; TRiP-alix/+*) (right image) are shown. Two fGSCs interconnected to multiple Nanos-positive cells via fusomes are seen in the anterior tip of the germarium in the right image. **(G)** Germarium from *control* female (*Bam-GAL4/+*) (left image) with Nanos-positive fGSCs with spectrosomes and germarium in which RNAi-mediated depletion of *alix* was performed using *Bam-GAL4* (*Bam-GAL4/TRiP-alix*) (right image) are shown. fGSCs with spectrosomes are seen in the anterior tip of the germaria. In (E-G) ovaries were fixed and stained with antibodies against Nanos (green) and hts-F (red). Scale bars represent 10 μm. **(H)** Graph showing the average percentages of germaria with normal or abnormal (linear or branched elongated) fusome morphologies in germaria of females of the genotypes in (E-G). *Nos-Gal4/+*, three independent experiments, n = 155 germaria; *Nos-Gal4/+; TRiP-alix/+*, three independent experiments, n = 168 germaria; *Dcr2/+; Nos-GAL4/+*, three independent experiments, n = 158 germaria; *Dcr2/+; Nos-GAL4/+; TRiP-alix/+*, three independent experiments, n = 139 germaria; *Bam-*

*GAL4*/+, three independent experiments, n = 174 germaria; *Bam-GAL4/TRiP-alix*, three independent experiments, n = 172 germaria. Data are presented as mean ± STD.
(TIF)

**S6 Fig. Stem cysts form in *alix* mutant germaria. (A)** Examples of *wild type*, $alix^1$ and $alix^3$ germaria stained for p-MAD (red) are shown. The *wild type* p-Mad-positive fGSC (red) is connected to a daughter cell CB via fusome (white). The $alix^1$ and $alix^3$ p-Mad-positive fGSCs (red) are connected chains of daughter cell via elongated fusomes. CC, cap cell. Scale bars represent 5μm. **(B-D)** Images showing Bam protein staining of *wild type*, $alix^1$ and $alix^3$ germaria. An fGSC-CB pair (B) or stem cysts (C-D) are outlined. Ovaries were stained with antibodies against Bam (green) and Cindr (red), and with Hoechst (blue). Scale bars represent 10 μm.
(TIF)

**S7 Fig. ALIX promotes abscission in *Drosophila* female germline stem cells. (A-B)** Shown are examples *wild type* germaria with normal fGSC morphologies: (i) a single fGSCs with a spectrosome (red, SP), (ii) fGSC-CB pairs undergoing cytokinesis with midbody rings (MR, green, arrows) and fused fusomes (red) and (iii) an fGSC-CB pair in late cytokinesis with a midbody (MB, green, arrowhead) and fusome with exclamation point morphology (red). **(C-D)** $alix^1$ mutant germaria show abnormal fGSC morphologies. Shown are fGSCs connected to more than one daughter cell in (iv) linear (C) or (v) branched (D) chains via midbody rings (green) and fusome (red). Cells interconnected are marked with asterisks. Ovaries in (A-D) were fixed and stained with antibodies against Cindr (green) and hts-F (red), with phalloidin to visualize F-actin (white) and with Hoechst (blue). Scale bars in (A-D) represent 5 μm. **(E)** Graph showing the average percentage of fGSCs with the indicated phenotypes from *wild type* and $alix^1$ mutant flies. *Wild type*, three independent experiments, n = 110, 30 germaria; $alix^1$, three independent experiments, n = 70, 29 germaria. The larger proportion of polyploid fGSCs in the $alix^1$ compared to the $alix^3$ mutant germaria might be explained by the fact that the $alix^1$ mutant flies were older than the $alix^3$ mutant females (7 days compared to 2–4 days old), allowing for more time for cleavage furrow regression. Data are presented as mean ± STD. See also S4 Table. **(F)** *Wild-type* germarium with normal fGSC morphologies. Shown are fGSCs with spectrosomes (hts-F, blue). **(G-H)** In $alix^1$ (G) and $alix^3$ (H) mutant germaria, bi- and multinucleate fGSCs can be detected. Nuclei of cells with more than one nucleus are marked with asterisks. Bi- and multi-nucleate phenotypes were mostly detected in flies older than four days and in some cases the midbody ring was still visible in the cell (H), indicating that it lost the connection to the plasma membrane. Ovaries in (F-H) were fixed and stained with antibodies against Cindr (green) and hts-F (blue), with rhodamine-phalloidin to visualize F-actin (red) and with Hoechst (white). Scale bars represent 10 μm. **(I)** Germarium from *control* female (*Nos-GAL4*/+) with fGSC-CB pair in abscission with an MB (arrow, green) dividing the fusome (red) (left image) and germarium with *alix-RNAi* expression (*Nos-GAL4/+; TRiP-alix/+*) (right image) are shown. In the right image an fGSC is interconnected to several daughter cells via MRs (arrows, green) and fusome (red). **(J)** Germarium from *control* female (*Dcr2/+; Nos-GAL4/+*) with fGSC-CB pair in abscission with an MB (arrow, green) dividing the fusome (red) (left image) and germarium with *alix-RNAi* and Dicer expression (*Dcr2/+; Nos-GAL4/+; TRiP-alix/+*) (right image) are shown. In the right image an fGSC is interconnected to several daughter cells via MRs (arrows, green) and fusome (red). **(K)** Germarium from *control* female (*Bam-GAL4*/+) (left image) and germarium in which RNAi-mediated depletion of *alix* was performed using *Bam-GAL4* (*Bam-GAL4/TRiP-alix*) (right image) are shown. fGSCs in both images display normal abscission (MR and MBs indicated with arrows, green). In (I-K) ovaries were fixed and stained with antibodies against Cindr (green) and hts-F (red). Scale bars represent 10 μm (full germaria) and 5 μm (enlarged images). **(L-N)** Shown in (L) is an fGSC-CB pair

in abscission (MB, arrow). In chains of cells interconnected to fGSCs (marked with asterisks and outlined) in *alix*$^3$ (M) and *alix*$^1$ (N) mutant germaria, MBs are detected (arrows), indicating abscission events of 2-cell cysts with MRs/RCs (arrowheads). Ovaries were fixed and stained with antibodies against Cindr (green, L-M) and hts-F (red, L-M and white, N), and with Hoechst (blue). F-actin is stained in red in N. Scale bars represent 5 μm.
(TIF)

**S8 Fig. ALIX controls abscission in *Drosophila* male germline stem cells. (A)** Western blot showing the lack of ALIX protein *alix*$^1$ and *alix*$^3$ mutant testes. Equal protein loading is validated by the levels of α-tubulin. **(B)** *Wild type* testis tip with mGSC-gonialblast (GB) pair (outlined) in cytokinesis interconnected by an MR (Cindr, green) and fusome (α-spectrin, red). **(C-D)** Testis tips in *alix*$^1$ and *alix*$^3$ mutants with mGSCs connected to chains of daughter cells (outlined) via MRs (green) and fusome (red). Testes in (B-D) were fixed and stained with antibodies against Cindr (green), α-spectrin (red) and FasIII (red), and with Hoechst (blue). Hubs are indicated with asterisks. Scale bars represent 5 μm. **(E)** Graph showing the average percentage of mGSCs with the indicated phenotypes. *Wild type*, three independent experiments, n = 166 mGSCs, 17 testes; *alix*$^1$, three independent experiments, n = 201 mGSCs, 17 testes; *alix*$^3$, three independent experiments, n = 119 mGSCs, 17 testes.
(TIF)

**S9 Fig. ALIX and Shrub localization at midbody rings during abscission in *Drosophila* female germline stem cells. (A-D)** Ovaries of 1–2 day-old *UASp-GFP-ALIX/+; Nanos-GAL4/+* (A-C) or *Nanos-GAL4*, *UASp-GFP-Shrub* (D) flies were dissected, fixed and stained with anti-α-tubulin (red), GFP Booster (green), anti-hts-F (white) and Hoechst (blue). Arrows indicate localization of GFP-ALIX to fusome plugs (A), MRs in G1/S (B, bar-shaped fusome), S phase (C, dumbbell-shaped fusome) and G2 (B, fusing fusome). Intercellular bridge MTs are present early when the fusome has plug morphology. At this point only weak GFP-Shrub signal is detected and in G2 phase GFP-Shrub is detected at an MR (D). Scale bars represent 5 μm.
(TIF)

**S10 Fig. ALIX and Shrub act together to control abscission in *Drosophila* female germline stem cells. (A-D)** Control EC with 4 RCs (arrows) to the oocyte (A) and *alix-RNAi* (B), *shrub-RNAi* (C) as well as *shrub-* and *alix-RNAi* (D) ECs with 5 RCs (arrows) to the oocyte. Ovaries were fixed and stained with phalloidin to visualize F-actin (white) and with Hoechst (blue). Scale bars represent 10 μm (left images) and 5 μm (right images). **(E)** Graph showing the average percentage of egg chambers (ECs) with 16, 32, more than 32 GCs, tumor phenotype and other phenotypes from control, *alix-RNAi*, *shrub-RNAi*, and *shrub & alix-RNAi* flies. Control, four independent experiments, n = 493 ECs; *alix-RNAi*, four independent experiments, n = 509 ECs; *shrub-RNAi*, four independent experiments, n = 231 ECs; *shrub & alix-RNAi*, 3 independent experiments, n = 215 ECs. **(F)** Graph showing the average percentages of germaria with the indicated phenotypes from *wild type*, *alix*$^1$, *shrub*$^{G5}$/+ and *shrub*$^{G5}$*/+; alix*$^1$ germaria. *Wild type*, four independent experiments, n = 29 germaria; *alix*$^1$, three independent experiments, n = 23 germaria; *shrub*$^{G5}$/+, three independent experiments, n = 27 germaria; *shrub*$^{G5}$*/+; alix*$^1$, three independent experiments, n = 33 germaria. **(G)** Graph showing the average number of stem cells per germarium from the *wild type*, *alix*$^1$, *shrub*$^{G5}$/+ and *shrub*$^{G5}$*/+; alix*$^1$ germaria in (F). *Wild type*, n = 80 fGSCs; *alix*$^1$, n = 50 fGSCs; *shrub*$^{G5}$/+, n = 70 fGSCs; *shrub*$^{G5}$*/+; alix*$^1$, n = 54 fGSCs. Data are presented as mean ± STD.
(TIF)

**S11 Fig. ALIX requires the interaction with Shrub to mediate abscission in *Drosophila* female germline stem cells. (A)** Images showing the localization of *wild type* GFP-ALIX, GFP-

ALIX-F198D or GFP-ALIX-I211D at MRs/MBs in fGSC-CB pairs or at MRs in stem cysts in germaria of the indicated genotypes. Ovaries were fixed and stained with antibodies against Cindr (red) and hts-F (white) and with Hoechst (blue). CC, cap cell. Scale bars represent 5 μm. **(B)** Graph showing the frequencies of stem cyst lengths for the indicated genotypes from the experiment in Fig. 7C. **(C)** Graph showing the average percentages of egg chambers with the indicated phenotypes in ovaries of females with the indicated genotypes from three independent experiments. *Nanos-GAL4/+*, n = 391 ECs; *Nanos-GAL4/UASp-GFP-ALIX*, 381 ECs; *Nanos-GAL4/UASp-GFP-ALIX-F198D*, 357 ECs; *Nanos-GAL4/UASp-GFP-ALIX-I211D*, 485 ECs; *alix*[1], 508 ECs; *Nanos-GAL4/UASp-GFP-ALIX; alix*[1], 392 ECs; *Nanos-GAL4/UASp-GFP-ALIX-F198D*; *alix*[1], 500 ECs; *Nanos-GAL4/UASp-GFP-ALIX-I211D; alix*[1], 378 ECs. Data are presented as mean ± STD.
(TIF)

**S1 Table. Percentages of follicle cells in *wild type*, *alix*[1] and *alix*[3] mutant egg chambers with one or more nuclei.**
(DOCX)

**S2 Table. Percentages of GFP-positive control and *alix*[3] mutant follicle cells with one or more nuclei.**
(DOCX)

**S3 Table. Number of *wild type* and *alix*[3] mutant germaria with normal versus abnormal fGSC phenotypes.**
(DOCX)

**S4 Table. Number of *wild type* and *alix*[1] mutant germaria with normal versus abnormal fGSC phenotypes.**
(DOCX)

## Acknowledgments

We thank members of the Stenmark laboratory for helpful discussions, DSHB for antibodies, BDSC and Exelixis at Harvard Medical School for fly stocks and the TRiP at Harvard Medical School (NIH/NIGMS R01-GM084947) for providing transgenic RNAi fly stocks used in this study. The confocal microscopy core facility at Oslo University Hospital is acknowledged for access to confocal microscopes.

## Author Contributions

Conceived and designed the experiments: KH ÅHE LM. Performed the experiments: KH ÅHE LM ALC CBS. Analyzed the data: KH ÅHE LM ALC CBS IPN HS. Contributed reagents/materials/analysis tools: JRH JM. Wrote the paper: KH. Performed statistical analyses: KL. Edited the manuscript: ÅHE LM ALC IPN KL HS.

## References

1. Glotzer M (2005) The molecular requirements for cytokinesis. Science 307: 1735–1739. doi: 10.1126/science.1096896 PMID: 15774750
2. Eggert US, Mitchison TJ, Field CM (2006) Animal cytokinesis: from parts list to mechanisms. Annu Rev Biochem 75: 543–566. doi: 10.1146/annurev.biochem.74.082803.133425 PMID: 16756502
3. Barr FA, Gruneberg U (2007) Cytokinesis: placing and making the final cut. Cell 131: 847–860. doi: 10.1016/j.cell.2007.11.011 PMID: 18045532
4. Green RA, Paluch E, Oegema K (2012) Cytokinesis in animal cells. Annu Rev Cell Dev Biol 28: 29–58. doi: 10.1146/annurev-cellbio-101011-155718 PMID: 22804577

5. Cabernard C (2012) Cytokinesis in Drosophila melanogaster. Cytoskeleton (Hoboken) 69: 791–809. doi: 10.1002/cm.21060

6. D'Avino P (2009) How to scaffold the contractile ring for a safe cytokinesis—lessons from Anillin-related proteins. J Cell Sci 122: 1071–1079. doi: 10.1242/jcs.034785 PMID: 19339546

7. Fededa JP, Gerlich DW (2012) Molecular control of animal cell cytokinesis. Nat Cell Biol 14: 440–447. doi: 10.1038/ncb2482 PMID: 22552143

8. Chen CT, Hehnly H, Doxsey SJ (2012) Orchestrating vesicle transport, ESCRTs and kinase surveillance during abscission. Nat Rev Mol Cell Biol 13: 483–488. doi: 10.1038/nrm3395 PMID: 22781903

9. Guizetti J, Schermelleh L, Mantler J, Maar S, Poser I, et al. (2011) Cortical constriction during abscission involves helices of ESCRT-III-dependent filaments. Science 331: 1616–1620. doi: 10.1126/science.1201847 PMID: 21310966

10. Elia N, Ott C, Lippincott-Schwartz J (2013) Incisive imaging and computation for cellular mysteries: lessons from abscission. Cell 155: 1220–1231. doi: 10.1016/j.cell.2013.11.011 PMID: 24315094

11. Elia N, Sougrat R, Spurlin TA, Hurley JH, Lippincott-Schwartz J (2011) Dynamics of endosomal sorting complex required for transport (ESCRT) machinery during cytokinesis and its role in abscission. Proc Natl Acad Sci U S A 108: 4846–4851. doi: 10.1073/pnas.1102714108 PMID: 21383202

12. Caballe A, Martin-Serrano J (2011) ESCRT machinery and cytokinesis: the road to daughter cell separation. Traffic 12: 1318–1326. doi: 10.1111/j.1600-0854.2011.01244.x PMID: 21722282

13. Carlton JG, Agromayor M, Martin-Serrano J (2008) Differential requirements for Alix and ESCRT-III in cytokinesis and HIV-1 release. Proc Natl Acad Sci U S A 105: 10541–10546. doi: 10.1073/pnas.0802008105 PMID: 18641129

14. Carlton JG, Martin-Serrano J (2007) Parallels between cytokinesis and retroviral budding: a role for the ESCRT machinery. Science 316: 1908–1912. doi: 10.1126/science.1143422 PMID: 17556548

15. Morita E, Sandrin V, Chung HY, Morham SG, Gygi SP, et al. (2007) Human ESCRT and ALIX proteins interact with proteins of the midbody and function in cytokinesis. Embo J 26: 4215–4227. doi: 10.1038/sj.emboj.7601850 PMID: 17853893

16. El Amine N, Kechad A, Jananji S, Hickson GR (2013) Opposing actions of septins and Sticky on Anillin promote the transition from contractile to midbody ring. J Cell Biol 203: 487–504. doi: 10.1083/jcb.201305053 PMID: 24217622

17. Mullins JM, Biesele JJ (1977) Terminal phase of cytokinesis in D-98s cells. J Cell Biol 73: 672–684. doi: 10.1083/jcb.73.3.672 PMID: 873994

18. Kechad A, Jananji S, Ruella Y, Hickson GR (2012) Anillin acts as a bifunctional linker coordinating midbody ring biogenesis during cytokinesis. Curr Biol 22: 197–203. doi: 10.1016/j.cub.2011.11.062 PMID: 22226749

19. Bassi ZI, Audusseau M, Riparbelli MG, Callaini G, D'Avino PP (2013) Citron kinase controls a molecular network required for midbody formation in cytokinesis. Proc Natl Acad Sci U S A 110: 9782–9787. doi: 10.1073/pnas.1301328110 PMID: 23716662

20. Green RA, Mayers JR, Wang S, Lewellyn L, Desai A, et al. (2013) The midbody ring scaffolds the abscission machinery in the absence of midbody microtubules. J Cell Biol 203: 505–520. doi: 10.1083/jcb.201306036 PMID: 24217623

21. Bastos RN, Barr FA (2010) Plk1 negatively regulates Cep55 recruitment to the midbody to ensure orderly abscission. J Cell Biol 191: 751–760. doi: 10.1083/jcb.201008108 PMID: 21079244

22. Lee HH, Elia N, Ghirlando R, Lippincott-Schwartz J, Hurley JH (2008) Midbody targeting of the ESCRT machinery by a noncanonical coiled coil in CEP55. Science 322: 576–580. doi: 10.1126/science.1162042 PMID: 18948538

23. Elia N, Fabrikant G, Kozlov MM, Lippincott-Schwartz J (2012) Computational model of cytokinetic abscission driven by ESCRT-III polymerization and remodeling. Biophys J 102: 2309–2320. doi: 10.1016/j.bpj.2012.04.007 PMID: 22677384

24. Yang D, Rismanchi N, Renvoise B, Lippincott-Schwartz J, Blackstone C, et al. (2008) Structural basis for midbody targeting of spastin by the ESCRT-III protein CHMP1B. Nat Struct Mol Biol 15: 1278–1286. doi: 10.1038/nsmb.1512 PMID: 18997780

25. Greenbaum MP, Iwamori T, Buchold GM, Matzuk MM (2011) Germ cell intercellular bridges. Cold Spring Harb Perspect Biol 3: a005850. doi: 10.1101/cshperspect.a005850 PMID: 21669984

26. Haglund K, Nezis IP, Stenmark H (2011) Structure and functions of stable intercellular bridges formed by incomplete cytokinesis during development. Commun Integr Biol 4: 1–9. doi: 10.4161/cib.4.1.13550 PMID: 21509167

27. Robinson DN, Cooley L (1996) Stable intercellular bridges in development: the cytoskeleton lining the tunnel. Trends Cell Biol 6: 474–479. doi: 10.1016/0962-8924(96)84945-2 PMID: 15157506

28. Lacroix B, Maddox AS (2012) Cytokinesis, ploidy and aneuploidy. J Pathol 226: 338–351. doi: 10.1002/path.3013 PMID: 21984283
29. Mathieu J, Cauvin C, Moch C, Radford SJ, Sampaio P, et al. (2013) Aurora B and cyclin B have opposite effects on the timing of cytokinesis abscission in Drosophila germ cells and in vertebrate somatic cells. Dev Cell 26: 250–265. doi: 10.1016/j.devcel.2013.07.005 PMID: 23948252
30. Iwamori T, Iwamori N, Ma L, Edson MA, Greenbaum MP, et al. (2010) TEX14 interacts with CEP55 to block cell abscission. Mol Cell Biol 30: 2280–2292. doi: 10.1128/MCB.01392-09 PMID: 20176808
31. de Cuevas M, Spradling AC (1998) Morphogenesis of the Drosophila fusome and its implications for oocyte specification. Development 125: 2781–2789. PMID: 9655801
32. Pepling ME, de Cuevas M, Spradling AC (1999) Germline cysts: a conserved phase of germ cell development? Trends Cell Biol 9: 257–262. doi: 10.1016/S0962-8924(99)01594-9 PMID: 10370240
33. Spradling A, Fuller MT, Braun RE, Yoshida S (2011) Germline stem cells. Cold Spring Harb Perspect Biol 3: a002642. doi: 10.1101/cshperspect.a002642 PMID: 21791699
34. Horne-Badovinac S, Bilder D (2005) Mass transit: epithelial morphogenesis in the Drosophila egg chamber. Dev Dyn 232: 559–574. doi: 10.1002/dvdy.20286 PMID: 15704134
35. Huynh JR, St Johnston D (2004) The origin of asymmetry: early polarisation of the Drosophila germline cyst and oocyte. Curr Biol 14: R438–449. doi: 10.1016/j.cub.2004.05.040 PMID: 15182695
36. Salzmann V, Chen C, Chiang CY, Tiyaboonchai A, Mayer M, et al. (2014) Centrosome-dependent asymmetric inheritance of the midbody ring in Drosophila germline stem cell division. Mol Biol Cell 25: 267–275. doi: 10.1091/mbc.E13-09-0541 PMID: 24227883
37. Sheng XR, Matunis E (2011) Live imaging of the Drosophila spermatogonial stem cell niche reveals novel mechanisms regulating germline stem cell output. Development 138: 3367–3376. doi: 10.1242/dev.065797 PMID: 21752931
38. Haglund K, Nezis IP, Lemus D, Grabbe C, Wesche J, et al. (2010) Cindr interacts with anillin to control cytokinesis in Drosophila melanogaster. Curr Biol 20: 944–950. doi: 10.1016/j.cub.2010.03.068 PMID: 20451383
39. Mathe E, Inoue YH, Palframan W, Brown G, Glover DM (2003) Orbit/Mast, the CLASP orthologue of Drosophila, is required for asymmetric stem cell and cystocyte divisions and development of the polarised microtubule network that interconnects oocyte and nurse cells during oogenesis. Development 130: 901–915. doi: 10.1242/dev.00315 PMID: 12538517
40. Brawley C, Matunis E (2004) Regeneration of male germline stem cells by spermatogonial dedifferentiation in vivo. Science 304: 1331–1334. doi: 10.1126/science.1097676 PMID: 15143218
41. Eikenes AH, Brech A, Stenmark H, Haglund K (2013) Spatiotemporal control of Cindr at ring canals during incomplete cytokinesis in the Drosophila male germline. Dev Biol 377: 9–20. doi: 10.1016/j.ydbio.2013.02.021 PMID: 23499247
42. Miyauchi C, Kitazawa D, Ando I, Hayashi D, Inoue YH (2013) Orbit/CLASP is required for germline cyst formation through its developmental control of fusomes and ring canals in Drosophila males. PLoS One 8: e58220. doi: 10.1371/journal.pone.0058220 PMID: 23520495
43. Yadlapalli S, Yamashita YM (2013) Chromosome-specific nonrandom sister chromatid segregation during stem-cell division. Nature 498: 251–254. doi: 10.1038/nature12106 PMID: 23644460
44. Hudson AM, Cooley L (2014) Methods for studying oogenesis. Methods 68: 207–217. doi: 10.1016/j.ymeth.2014.01.005 PMID: 24440745
45. Ni JQ, Zhou R, Czech B, Liu LP, Holderbaum L, et al. (2011) A genome-scale shRNA resource for transgenic RNAi in Drosophila. Nat Methods 8: 405–407. doi: 10.1038/nmeth.1592 PMID: 21460824
46. Yan D, Neumuller RA, Buckner M, Ayers K, Li H, et al. (2014) A regulatory network of Drosophila germline stem cell self-renewal. Dev Cell 28: 459–473. doi: 10.1016/j.devcel.2014.01.020 PMID: 24576427
47. Lilly MA, de Cuevas M, Spradling AC (2000) Cyclin A associates with the fusome during germline cyst formation in the Drosophila ovary. Dev Biol 218: 53–63. doi: 10.1006/dbio.1999.9570 PMID: 10644410
48. Hawkins NC, Thorpe J, Schupbach T (1996) Encore, a gene required for the regulation of germ line mitosis and oocyte differentiation during Drosophila oogenesis. Development 122: 281–290. PMID: 8565840
49. Ables ET, Drummond-Barbosa D (2013) Cyclin E controls Drosophila female germline stem cell maintenance independently of its role in proliferation by modulating responsiveness to niche signals. Development 140: 530–540. doi: 10.1242/dev.088583 PMID: 23293285
50. Deng W, Lin H (1997) Spectrosomes and fusomes anchor mitotic spindles during asymmetric germ cell divisions and facilitate the formation of a polarized microtubule array for oocyte specification in Drosophila. Dev Biol 189: 79–94. doi: 10.1006/dbio.1997.8669 PMID: 9281339

51. Hsu HJ, LaFever L, Drummond-Barbosa D (2008) Diet controls normal and tumorous germline stem cells via insulin-dependent and-independent mechanisms in Drosophila. Dev Biol 313: 700–712. doi: 10.1016/j.ydbio.2007.11.006 PMID: 18068153

52. McCullough J, Colf LA, Sundquist WI (2013) Membrane fission reactions of the mammalian ESCRT pathway. Annu Rev Biochem 82: 663–692. doi: 10.1146/annurev-biochem-072909-101058 PMID: 23527693

53. Guizetti J, Gerlich DW (2012) ESCRT-III polymers in membrane neck constriction. Trends Cell Biol 22: 133–140. doi: 10.1016/j.tcb.2011.11.007 PMID: 22240455

54. McCullough J, Fisher RD, Whitby FG, Sundquist WI, Hill CP (2008) ALIX-CHMP4 interactions in the human ESCRT pathway. Proc Natl Acad Sci U S A 105: 7687–7691. doi: 10.1073/pnas.0801567105 PMID: 18511562

55. Matias NR, Mathieu J, Huynh JR (2015) Abscission is regulated by the ESCRT-III protein Shrub in Drosophila germline stem cells. PLoS Genet. 11: e1004653.

56. Lindas AC, Karlsson EA, Lindgren MT, Ettema TJ, Bernander R (2008) A unique cell division machinery in the Archaea. Proc Natl Acad Sci U S A 105: 18942–18946. doi: 10.1073/pnas.0809467105 PMID: 18987308

57. Samson RY, Obita T, Freund SM, Williams RL, Bell SD (2008) A role for the ESCRT system in cell division in archaea. Science 322: 1710–1713. doi: 10.1126/science.1165322 PMID: 19008417

58. Lindas AC, Bernander R (2013) The cell cycle of archaea. Nat Rev Microbiol 11: 627–638. doi: 10.1038/nrmicro3077 PMID: 23893102

59. Spitzer C, Schellmann S, Sabovljevic A, Shahriari M, Keshavaiah C, et al. (2006) The Arabidopsis elch mutant reveals functions of an ESCRT component in cytokinesis. Development 133: 4679–4689. doi: 10.1242/dev.02654 PMID: 17090720

60. McMurray MA, Stefan CJ, Wemmer M, Odorizzi G, Emr SD, et al. (2011) Genetic interactions with mutations affecting septin assembly reveal ESCRT functions in budding yeast cytokinesis. Biol Chem 392: 699–712. doi: 10.1515/BC.2011.091 PMID: 21824003

61. Renshaw MJ, Liu J, Lavoie BD, Wilde A (2014) Anillin-dependent organization of septin filaments promotes intercellular bridge elongation and Chmp4B targeting to the abscission site. Open Biol 4: 130190. doi: 10.1098/rsob.130190 PMID: 24451548

62. Mondal G, Rowley M, Guidugli L, Wu J, Pankratz VS, et al. (2012) BRCA2 localization to the midbody by filamin A regulates cep55 signaling and completion of cytokinesis. Dev Cell 23: 137–152. doi: 10.1016/j.devcel.2012.05.008 PMID: 22771033

63. Sagona AP, Nezis IP, Pedersen NM, Liestol K, Poulton J, et al. (2010) PtdIns(3)P controls cytokinesis through KIF13A-mediated recruitment of FYVE-CENT to the midbody. Nat Cell Biol 12: 362–371. doi: 10.1038/ncb2036 PMID: 20208530

64. Neto H, Kaupisch A, Collins LL, Gould GW (2013) Syntaxin 16 is a master recruitment factor for cytokinesis. Mol Biol Cell 24: 3663–3674. doi: 10.1091/mbc.E13-06-0302 PMID: 24109596

65. Pires R, Hartlieb B, Signor L, Schoehn G, Lata S, et al. (2009) A crescent-shaped ALIX dimer targets ESCRT-III CHMP4 filaments. Structure 17: 843–856. doi: 10.1016/j.str.2009.04.007 PMID: 19523902

66. Kim J, Sitaraman S, Hierro A, Beach BM, Odorizzi G, et al. (2005) Structural basis for endosomal targeting by the Bro1 domain. Dev Cell 8: 937–947. doi: 10.1016/j.devcel.2005.04.001 PMID: 15935782

67. Wemmer M, Azmi I, West M, Davies B, Katzmann D, et al. (2011) Bro1 binding to Snf7 regulates ESCRT-III membrane scission activity in yeast. J Cell Biol 192: 295–306. doi: 10.1083/jcb.201007018 PMID: 21263029

68. Bissig C, Gruenberg J (2013) ALIX and the multivesicular endosome: ALIX in Wonderland. Trends Cell Biol 24: 19–25. doi: 10.1016/j.tcb.2013.10.009 PMID: 24287454

69. Dobro MJ, Samson RY, Yu Z, McCullough J, Ding HJ, et al. (2013) Electron cryotomography of ESCRT assemblies and dividing Sulfolobus cells suggests that spiraling filaments are involved in membrane scission. Mol Biol Cell 24: 2319–2327. doi: 10.1091/mbc.E12-11-0785 PMID: 23761076

70. Carlton JG, Caballe A, Agromayor M, Kloc M, Martin-Serrano J (2012) ESCRT-III governs the Aurora B-mediated abscission checkpoint through CHMP4C. Science 336: 220–225. doi: 10.1126/science.1217180 PMID: 22422861

71. Capalbo L, Montembault E, Takeda T, Bassi ZI, Glover DM, et al. (2012) The chromosomal passenger complex controls the function of endosomal sorting complex required for transport-III Snf7 proteins during cytokinesis. Open Biol 2: 120070. doi: 10.1098/rsob.120070 PMID: 22724069

72. McKearin D, Ohlstein B (1995) A role for the Drosophila bag-of-marbles protein in the differentiation of cystoblasts from germline stem cells. Development 121: 2937–2947. PMID: 7555720

**73.** Ohlstein B, McKearin D (1997) Ectopic expression of the Drosophila Bam protein eliminates oogenic germline stem cells. Development 124: 3651–3662. PMID: 9342057

**74.** Sweeney NT, Brenman JE, Jan YN, Gao FB (2006) The coiled-coil protein shrub controls neuronal morphogenesis in Drosophila. Curr Biol 16: 1006–1011. doi: 10.1016/j.cub.2006.03.067 PMID: 16713958

**75.** Luo L, Chai PC, Cai Y (2013) Immunostaining of germline stem cells and the niche in Drosophila ovaries. Methods Mol Biol 1035: 1–7. doi: 10.1007/978-1-62703-508-8_1 PMID: 23959977

**76.** D'Avino PP, Archambault V, Przewloka MR, Zhang W, Laue ED, et al. (2009) Isolation of protein complexes involved in mitosis and cytokinesis from Drosophila cultured cells. Methods Mol Biol 545: 99–112. doi: 10.1007/978-1-60327-993-2_6 PMID: 19475384

**77.** Venken KJ, Carlson JW, Schulze KL, Pan H, He Y, et al. (2009) Versatile P[acman] BAC libraries for transgenesis studies in Drosophila melanogaster. Nat Methods 6: 431–434. doi: 10.1038/nmeth.1331 PMID: 19465919

**78.** Rothwell WF, Sullivan W (2007) Fixation of Drosophila embryos. CSH Protoc 2007: pdb prot4827

**79.** Woodruff RI, Tilney LG (1998) Intercellular bridges between epithelial cells in the Drosophila ovarian follicle: a possible aid to localized signaling. Dev Biol 200: 82–91. doi: 10.1006/dbio.1998.8948 PMID: 9698458

**80.** Airoldi SJ, McLean PF, Shimada Y, Cooley L (2011) Intercellular protein movement in syncytial Drosophila follicle cells. Journal of Cell Science 124: 4077–4086. doi: 10.1242/jcs.090456 PMID: 22135360
